# Post‐translational regulation of human D‐3‐phosphoglycerate dehydrogenase in Alzheimer's disease

**DOI:** 10.1002/pro.70505

**Published:** 2026-02-19

**Authors:** Elena Zerbini, Daniele Riva, Elisa Maffioli, Gabriella Tedeschi, Silvia Sacchi, Loredano Pollegioni

**Affiliations:** ^1^ Department of Biotechnology and Life Sciences University of Insubria Varese Italy; ^2^ Department of Veterinary Medicine and Animal Science University of Milano Lodi Italy; ^3^ Present address: Department of Biosciences University of Milano Milano Italy

**Keywords:** acetylation, enzyme regulation, PHGDH, phosphorylation, serine

## Abstract

Emerging evidence suggests that sex‐specific differences in L‐serine (L‐Ser) metabolism play a key role in Alzheimer's disease (AD). While disruptions in amino acid balance are well known, recent findings point to a dimorphic regulation of the serine biosynthetic pathway. To explore this, we examined post‐translational modifications (PTMs) of D‐3‐phosphoglycerate dehydrogenase (PHGDH)—the rate‐limiting enzyme for de novo L‐Ser synthesis—as a potentialmechanism underlying this difference. PHGDH was immunoprecipitated from hippocampal tissue of healthy and AD‐affected males and females and analyzed by mass spectrometry. Five phosphorylation sites (S55, T60, T78, S383, and S473) were shared across all groups, but a unique deacetylation at K289 appeared exclusively in AD males. Functional assays using recombinant PHGDH variants revealed that changes at solvent‐exposed sites (K289, S383, and S473) reduced solubility, while phosphomimetic substitutions at S55 and T78 within the catalytic cleft strongly impaired activity. Notably, mimicking acetylation at K289 improved protein stability. Overall, these PTMs act both as subtle modulators and as on/off switches, fine‐tuning PHGDH function and potentially contributing to sex‐dependent metabolic vulnerability in AD.

## 1 INTRODUCTION

Memory deficits, cognitive impairment, and motor dysfunction are characteristics of Alzheimer's disease (AD), the most frequent cause of dementia worldwide (Qiu et al., 2009). This progressive, chronic neurodegenerative disorder has been known to compromise the functionality of several brain regions; among them, the hippocampus, a critical area for learning and memory (Kapogiannis & Mattson, 2011), is the one contributing most to the widely known AD‐related symptomatology. Despite several decades of studies, AD still remains a largely idiopathic disorder displaying a complex and multifactorial nature. A remarkable overlap has been recognized between the early onset (familial) form, caused by autosomal dominant mutations in genes codifying the amyloid precursor protein and presenilin, and the late‐onset (sporadic) form, associated with a combination of genetic background and environmental agents (Barber, 2012; Butterfield & Halliwell, 2019). Concerning risk factors, female sex has been reported to largely impact the pathogenesis of AD: compared to men, women have almost doubled chances to develop the disease during their lifetime (Seshadri et al., 1997). In this regard, the very recent proposal of alterations of the L‐serine (L‐Ser) and D‐serine (D‐Ser) biosynthetic pathways as compensatory mechanisms to counteract N‐methyl‐D‐aspartate receptors (NMDAR) hypofunction during early AD in females only (Maffioli et al., 2022) might suggest an additional source of the differences observed between sexes in the neurodegenerative progression.

Based on a consistent body of evidence (Chen et al., 2022; Le Douce et al., 2020; Yan et al., 2020), disequilibria in brain L‐Ser homeostasis are currently proposed to be intimately connected to AD: L‐Ser and its synthesis through the phosphorylated pathway (PP) are essential for maintaining steady‐state levels of D‐Ser, the main endogenous co‐agonist of synaptic NMDARs (Yang et al., 2010). In the adult central nervous system, the PP is of paramount importance to provide adequate L‐Ser levels that, otherwise, would not be guaranteed due to the extremely poor diffusion of the amino acid across the blood–brain barrier (Maugard et al., 2021). Notably, the brain permeability to L‐Ser increases during mouse development due to the activity of the amino acid transporter Slc38a5 (Radzishevsky et al., 2023). Primarily expressed and active in glial cells, the PP is a short metabolic pathway rerouting the glycolytic intermediate D‐3‐phosphoglycerate (3‐PG) toward the L‐Ser production through the coordinated action of three enzymes, 3‐phosphoglycerate dehydrogenase (PHGDH, EC 1.1.1.95), phosphoserine aminotransferase (PSAT, EC 2.6.1.52) and phosphoserine phosphatase (PSP, EC 3.1.3.3) (Murtas et al., 2020). Remarkably, the PP's enzymes have been recently shown to be organized in a transient metabolic assembly, named “serinosome,” composed of multiple copies of the three proteins (Rabattoni et al., 2023). Once altered—either slowed down by the impaired glucose intake (Le Douce et al., 2020) or upregulated, likely in response to amyloid beta plaques‐induction (Wu et al., 2004)—the PP yields insufficient or abnormal levels of D‐Ser that may contribute to the AD‐related NMDARs dysfunctions. Therefore, elucidating the mechanisms that regulate the PP, particularly those affecting PHGDH activity (Murtas et al., 2024), may shed light on the molecular bases of sex‐dependent vulnerability to AD.

To date, experimental evidence on the regulatory mechanisms controlling L‐Ser biosynthesis remains scarce. Beyond a bidirectional control between PP and glycolysis (Chaneton et al., 2012; Hitosugi et al., 2012; Jin et al., 2020; Ye et al., 2012), an increasing number of studies have identified post‐translational modifications (PTMs), mainly in cancer cells and tissues, as key modulators of the activity, stability, and compartmentalization of PHGDH, the enzyme catalyzing the first and rate‐limiting step of L‐Ser biosynthesis (Ma et al., 2013; Ma et al., 2021; Wang et al., 2020). Understanding whether similar regulatory mechanisms occur in physiological or neurodegenerative contexts is crucial to define the broader significance of PHGDH modulation. Accordingly, this study is aimed at identifying PHGDH PTMs in hippocampal tissues of AD‐affected and healthy individuals (males and females) by mass spectrometry analysis and to characterize protein variants mimicking the detected PTMs to assess how they affect the enzyme's biochemical and cellular properties as well as the rate of L‐Ser synthesis through the PP.

## 2 RESULTS

### 2.1 Immunoprecipitation of PHGDH from human hippocampus

**FIGURE 1:**
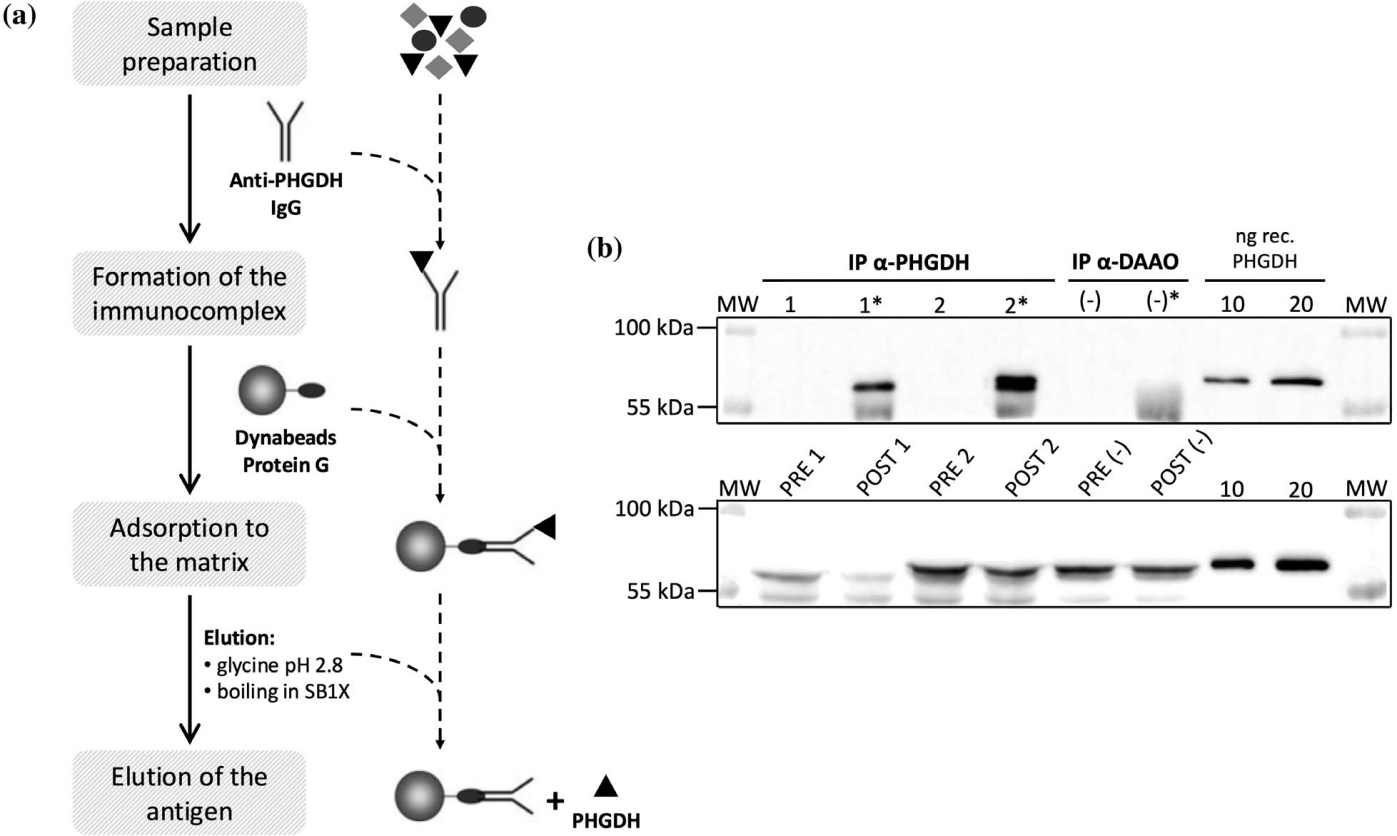
Immunoprecipitation of PHGDH from the human hippocampus. (a) Scheme of the IP protocol for the isolation of PHGDH from hippocampal samples. (b) Western blot analysis of immunoprecipitated samples. Sample 1 corresponds to hippocampal lysate, Sample 2 to the positive control, and sample (−) to the negative control. Upper panel (IP samples): Fractions eluted in 50 mM glycine, pH 2.8 (lanes 1, 2, and [−]) and in SB1X (lanes 1*, 2*, and (−)*). Lower panel (PRE and POST IP samples): 30 μL of each sample before IP (lanes PRE 1, PRE 2, and PRE (−)) and after the incubation with the antibody and the Dynabeads Protein G (lanes POST 1, POST 2, and POST [−]).

In order to identify the PTMs of PHGDH in the human brain of healthy and AD patients, the protein was isolated by immunoprecipitation (IP) using an anti‐PHGDH antibody and untargeted and targeted MS/MS analysis was performed. Endogenous PHGDH levels in the analyzed post‐mortem hippocampi of healthy and AD‐affected subjects were determined by Western blot analyses in a previous work (Maffioli et al., 2022) carried out on the same samples used here; a higher level was detected in the pathological tissue (Table S1). The IP was a challenging issue due to the low amount of endogenous PHGDH in the human samples. Therefore, the IP strategy was optimized by evaluating different amounts of sample, different commercial polyclonal antibodies, and the addition to tissue lysates of fixed amounts of the recombinant PHGDH as a positive control. The direct IP procedure—binding of the antibody to the bead support, Dynabeads protein G, addition of tissue lysates for immuno‐complex formation and elution of the protein of interest—resulted in poor IP yields. Better results were obtained by adopting an indirect method: lysate samples were added with the antibody, incubated overnight at 4°C and the bead matrix was added for immuno‐complex capturing. By using the optimized PHGDH‐IP protocol based on an antibody (Ab):antigen (Ag) molar ratio ~ 3:1 (Figure 1a), the protein was recovered in sufficient amounts from hippocampal samples of both healthy and AD‐affected subjects for subsequent analyses (Figure 1b). Western blot analyses show that the protein was largely detected in SB1X‐eluted fraction, whereas it was barely detectable in the glycine‐eluted one, suggesting a strong and stable association of PHGDH within the immuno‐complex. The densitometric evaluation of signals corresponding to PHGDH in lysates before (PRE‐IP sample) and after (POST‐IP sample) the immunoprecipitation indicated a mean IP yield of ~65%, corresponding to a maximal theoretical recovery of ~100 ng PHGDH starting from 250 μg of total proteins.

### 2.2 Identification of PTMs by MS/MS analyses on immunoprecipitated PHGDH

nLC‐MS/MS analyses were performed for the identification of PTMs of PHGDH isolated from hippocampal samples of female and male healthy and AD‐affected subjects (CTRF, healthy females; CTRM, healthy males; ADF, AD‐affected females; ADM, AD‐affected males) upon IP enrichment. Due to the limited amount of available samples, the analyses focused on two PTMs: phosphorylation and acetylation. Each sample was analyzed first by an untargeted MS/MS approach to identify the modified residues, followed by a targeted analysis to confirm the results.

**FIGURE 2:**
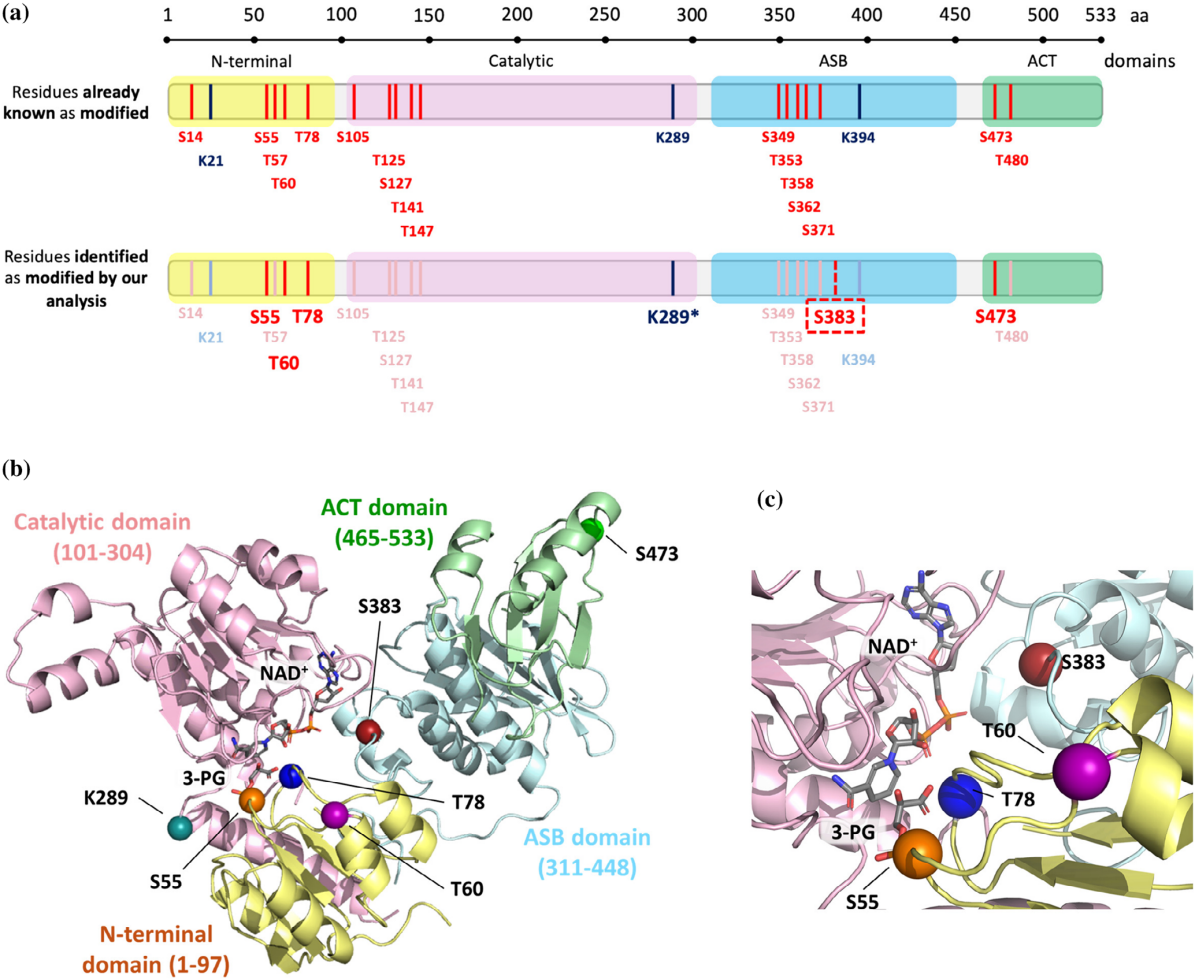
Residues modified by phosphorylation or acetylation in PHGDH from the human hippocampus. (a) Schematic representation of PHGDH's amino acid sequence, where phosphorylated and acetylated sites are indicated by red/pink or blue/light blue vertical lines, respectively. In particular, the two schemes show the comparison between the residues already known as modified in literature (scheme above) and those confirmed (red or blue vertical lines for phosphorylation and acetylation, respectively) or newly found (dashed vertical line) as modified by our analysis (scheme below). Note, the acetylation on K289 was not detected in AD‐affected males. (b,c) Structural model of the monomeric PHGDH. (b) Full‐length chain monomer built by superpositioning the experimentally solved structure (PDB 2G76, corresponding to the N‐terminal part) with the AlphaFold predicted one (AF‐O43175‐F1). The PHGDH structure PDB ID 6CWA was used as a template to add 3‐PG and NAD^+^ (represented in sticks with carbon, nitrogen, oxygen, and phosphate atoms colored in cyan, blue, red, and orange, respectively). Modified residues resulting from targeted MS/MS analysis are displayed as colored spheres (orange, purple, blue, firebrick, green, and deep teal for S55, T60, T78, S383, S473, and K289, respectively). (c) Zoom showing the reciprocal position of phosphorylated S55, T60, T78, and S383 compared to those of the cofactor NAD^+^ and the substrate 3‐PG at the level of the active cleft (N‐terminal and catalytic domains).

A prediction of putative phosphorylation and acetylation sites in PHGDH is reported in Table S2. The MS/MS analyses identified five residues (S55, T60, T78, S383, and S473) phosphorylated in each data set (Figures 2, S1, S2, and Table 1). Notably, among the modified residues, S383 has never been described as phosphorylated until now. To date, experimental evidence on PHGDH PTMs mainly derives from studies on tumor tissues or cell lines, where it is known to play a relevant role in cancer progression (Ma et al., 2013; Ma et al., 2021; Wang et al., 2020). In contrast, our analyses provide the first characterization of the protein PTMs in the human brain (of healthy and AD‐affected individuals). The presence of phosphorylation in all groups weakened the hypothesis of phosphorylation as a selective regulatory mechanism of PHGDH related to AD or sex. Due to the very limited amount of human material available, it was not possible to perform additional mass spectrometry analyses that could provide information on the degree of modification and site occupancy of each phosphorylated residue.

**TABLE 1:** Targeted MS/MS analysis of PHGDH.

Phosphorylation							
Residue	In this study				In previous studies		
	CTRF	CTRM	ADF	ADM	Phosphorylated	Kinase	References
S55	×	×	×	×	Yes	PKCζ	Santamaria et al., 2011; Ma et al., 2013; Mertins et al., 2013; Shiromizu et al., 2013
T60	×	×	×	×	Yes	Plk1	Santamaria et al., 2011; Shiromizu et al., 2013
T78	×	×	×	×	Yes	PKCζ	Ruse et al., 2008; Han et al., 2010; Ma et al., 2013; Mertins et al., 2013; Shiromizu et al., 2013; Zhou et al., 2013; Bian et al., 2014; Britton et al., 2014; Mertins et al., 2014; Sharma et al., 2014; Stuart et al., 2015
S383	×	×	×	×	No	–	–
S473	×	×	×	×	Predicted	Putative ATM/ATR/DNA‐PK kinase substrate	PhosphoSitePlus (https://www.phosphosite.org/curatedInfoAction.action?record=3358946)

Acetylation							
Residue	In this study				In previous studies		
	CTRF	CTRM	ADF	ADM	Acetylated	Acetyltransferase	References
K289	×	×	×		No	Unknown	Wang et al., 2020; Dai et al., 2023

*Note*: List of PHGDH residues identified as phosphorylated or acetylated in our targeted analyses of hippocampal samples from CTRF (healthy females), CTRM (healthy males), ADF (AD‐affected females), and ADM (AD‐affected males). Where available, our findings are compared with evidence from previous studies. Notably, residue S383 was neither experimentally detected nor predicted to be phosphorylated.

Furthermore, the targeted MS/MS analysis identified the K289 residue as acetylated in all samples except for AD‐affected males (Table 1).

### 2.3 Recombinant expression and purification of the PHGDH variants

In order to evaluate the effect of phosphorylation and acetylation at the identified sites in PHGDH, recombinant variants carrying an aspartate (to mimic phosphorylation) or a glutamine (to mimic acetylation) were produced, as well as the corresponding variants carrying a neutral alanine (alanine scanning for phosphorylation) or an arginine for positive charge conservation (for lysine acetylation). All the PHGDH variants were purified with a purity degree >90% (Figure S3). The highest expression levels were obtained for T60D (~22 mg/L), T78A (~24 mg/L), S55A (~15 mg/L), and T60A and S55D (~6 mg/L), see Table S3. Noteworthy, the alanine substitution at positions 383 and 473 in the C‐terminal region of PHGDH generated variant proteins prone to aggregation at 4°C; for this reason, after thawing these proteins were kept at room temperature for the following biochemical characterization. This suggests a putative role of the S383 and S473 residues on the overall protein stability.

### 2.4 Structural properties and stability of the PHGDH variants

**FIGURE 3:**
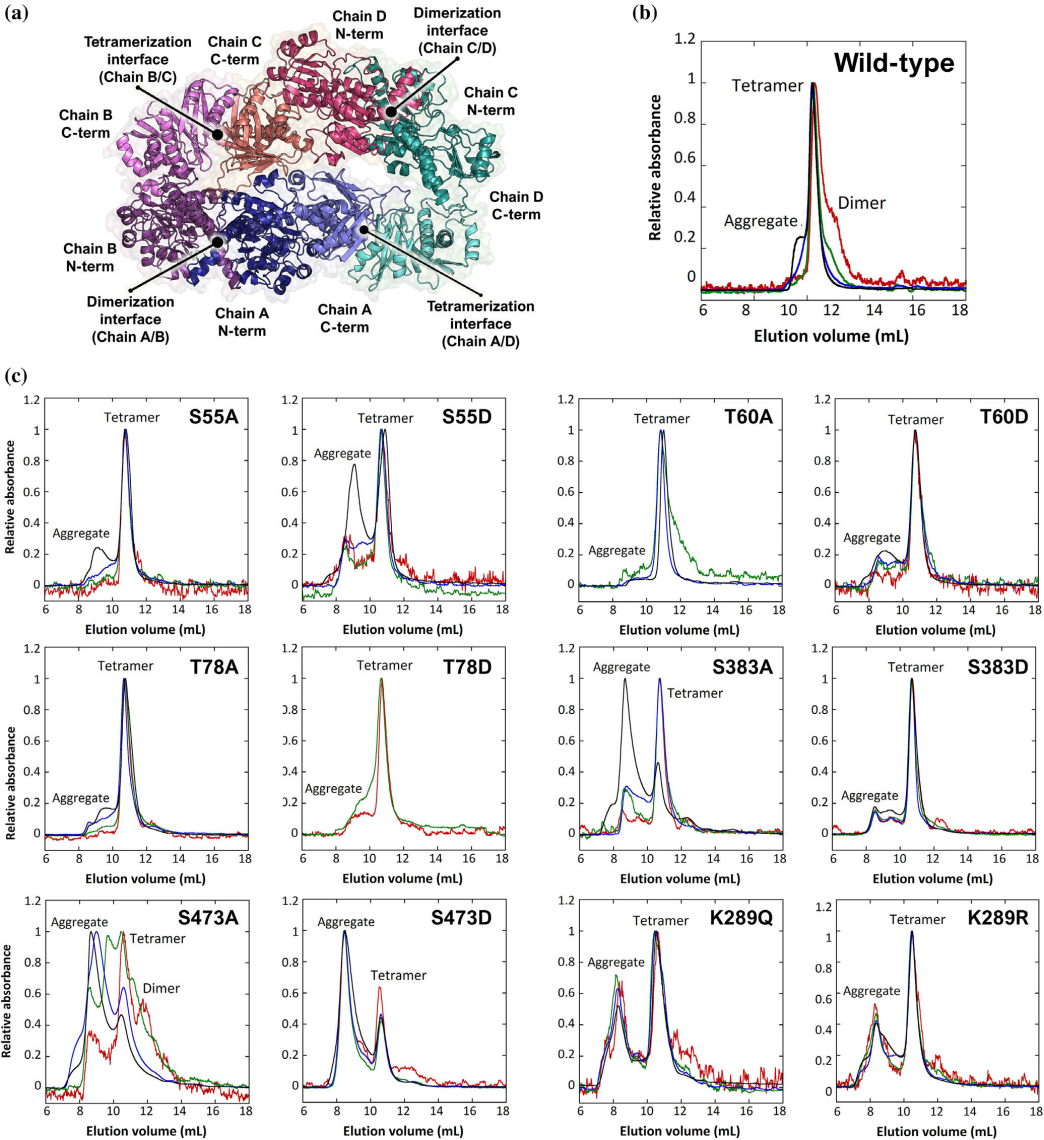
Quaternary structure of human PHGDH. (a) Putative quaternary structure of human PHGDH as determined by AlphaFold‐Multimer (Riva et al., 2024). (b, c) SEC chromatograms of PHGDH wild‐type and phosphorylation/acetylation variants (S55A/D, T60/D, T78A/D, S383A/D, S473/D, and K289Q/R) at different concentrations (0.33 mg/mL [red], 1 mg/mL [green], 3.33 mg/mL [blue], and 10 mg/mL [black]).

The oligomeric state—and thus the quaternary conformational stability—of the PHGDH variants was evaluated by size‐exclusion chromatography (SEC) across a range of protein concentrations (0.33, 1, 3.33, and 10 mg/mL) and compared to the wild‐type enzyme (Figure 3, top left panel). At 10 mg/mL, substitutions within the N‐terminal region did not markedly affect tetramer formation, although the presence of a left shoulder suggested early aggregation, indicating partial destabilization of the native fold. Among the analyzed variants, the S55D substitution produced the most evident change in oligomeric state, yielding a broadened peak with ~40% of the protein eluting as high‐molecular‐weight aggregates (Figure 3). SEC analysis could not be extended to high concentrations for the T78D variant due to protein precipitation during concentration, further suggesting reduced solubility. Notably, substitutions at the C‐terminal region—irrespective of whether they mimicked phosphorylation (Asp) or were charge‐neutral (Ala)—led to aggregation even below 10 mg/mL, underscoring the crucial contribution of the C‐terminal residues to long‐range stabilization of the PHGDH tetrameric architecture. At concentrations <3.33 mg/mL, variants at positions 383 and 473 remained largely tetrameric, although with detectable aggregation (<20%), confirming increased aggregation propensity. Strikingly, the S473D substitution consistently favored aggregation over native oligomerization at all concentrations tested (79% aggregate), indicating strong destabilization of intersubunit packing. Finally, the PHGDH K289 acetylation‐mimicking variants (K289Q and K289R) formed tetramers but also accumulated substantial aggregates across all conditions, further supporting the role of this site in structural stabilization rather than oligomeric switching (Figure 3).

Regarding the thermal stability, the melting temperature (T_m_) of the wild‐type protein and the PHGDH variants differed slightly: the most significant decrease (ΔT_m_ = −4.7°C) was observed for the T60A variant, and the maximum increase (ΔT_m_ = 3.0°C) for the S55D PHGDH (Table S4 and Figure S4).

Concerning the conformational studies, the far‐UV spectra for all the variants resembled the one for the wild‐type PHGDH, with some differences in the intensity of the signal that we suggest arising from partial protein precipitation (e.g., for T60A and T78D variants, Figure S5, right). The estimation of the secondary structure elements (Table S4 and Figure S5, left) indicated a similar content in secondary elements for all the variants compared to the wild‐type PHGDH, with the exception of the K289Q PHGDH variants.

In conclusion, the introduced substitutions do not affect the conformation of PHGDH to a significant extent, resulting in local alterations that mainly affect solubility and the stability of the homotetrameric state.

### 2.5 Kinetic parameters of the PHGDH variants

**FIGURE 4:**
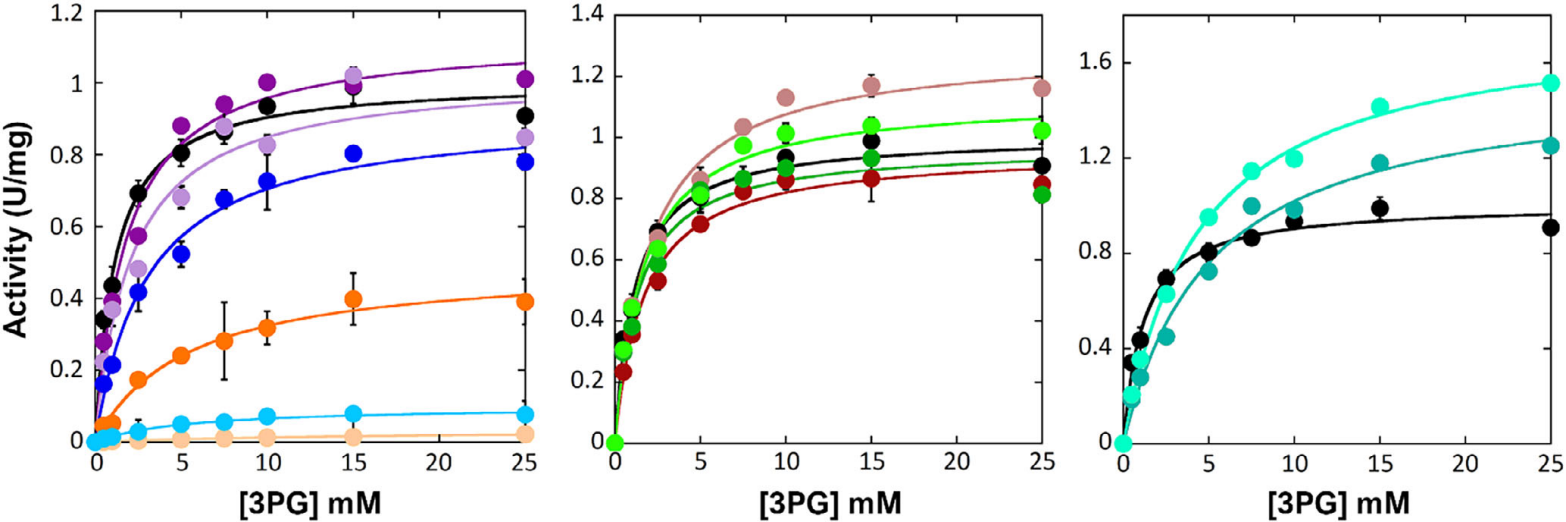
Kinetics of the forward reaction for wild‐type and variants of PHGDH. Kinetics were recorded in standard conditions at 37°C in 25 mM HEPES, pH 7.0. Left: Residues at the N‐terminal end of PHGDH (wild‐type [black], S55A [orange], S55D [pale orange], T60A [dark purple], T60D [lavender], T78A [blue], T78D [light blue]); center: Variants at residues at the C‐terminal end of PHGDH (wild‐type [black], S383A [firebrick], S383D [salmon], S473A [dark green], S473D [light green]); right: PHGDH variants mimicking the acetylation (wild‐type [black], K289Q [deep teal], K289R [aquamarine]). Data represent mean ± SD from independent experiments (*n* = 3).

The kinetic parameters of the PHGDH variants were assessed using a spectrophotometric standard coupled assay (Murtas et al., 2021; Murtas et al., 2020), monitoring the NADH production at 340 nm with the physiological substrate 3PG. For all PHGDH variants, the activity dependence on substrate concentration followed a Michaelis–Menten hyperbolic behavior, with no evidence for a sigmoidal behavior (Figure 4). Serine‐to‐alanine substitutions at positions 383 and 473 within the regulatory domains had no significant effect on k_cat_. However, replacing these residues with an aspartate (S383D and S473D variants) resulted in a slight increase in k_cat_ of around 28% and 13%, respectively, compared to the wild‐type PHGDH (Table 2). Actually, the k_cat_ for the S55D and T78D was 25 and 10‐fold lower than the one for wild‐type PHGDH, respectively: the presence of a negatively charged group close to the substrate hampered the enzyme's activity, likely due to electrostatic repulsion as indicated by the increase in K_M_ value. Moreover, both S55A and T78A substitutions resulted in a slight decrease in the turnover number. Interestingly, the substitutions at residue T60, even if belonging to the substrate and cofactor binding domain, did not yield a significant alteration in the k_cat_ value. Overall, the K_M_ value of all the variants was higher compared to the wild‐type PHGDH, and this resulted in a lower kinetic efficiency: a figure 435‐ and 44‐fold lower compared to the PHGDH wild‐type was evident for the S55D and T78D variants, respectively.

**TABLE 2:** Kinetic parameters of wild‐type and variants of human PHGDH.

Variants	k_cat_ (s^−1^)	K_M_ (mM)	k_cat_/K_M_ (mM·s^−1^)
Wild‐type	1.00 ± 0.02	1.2 ± 0.1	0.83
S55A	0.50 ± 0.03	5.3 ± 0.9	0.09
S55D	0.04 ± 0.01	24.7 ± 8.2	0.002
T60A	1.13 ± 0.04	1.8 ± 0.3	0.62
T60D	1.02 ± 0.07	2.1 ± 0.6	0.49
T78A	0.92 ± 0.04	3.0 ± 0.4	0.30
T78D	0.10 ± 0.01	5.2 ± 1.0	0.02
S383A	0.96 ± 0.03	1.7 ± 0.2	0.56
S383D	1.29 ± 0.04	2.0 ± 0.2	0.64
S473A	0.97 ± 0.04	1.3 ± 0.3	0.74
S473D	1.13 ± 0.03	1.6 ± 0.2	0.70
K289Q	1.49 ± 0.08	5.0 ± 0.7	0.30
K289R	1.78 ± 0.04	4.4 ± 0.3	0.41

*Note*: The kinetic parameters in the forward direction were determined at 37°C in 25 mM HEPES, pH 7.0, under standard conditions (Riva et al., 2024); values are mean ± SEM of three measurements.

**FIGURE 5:**
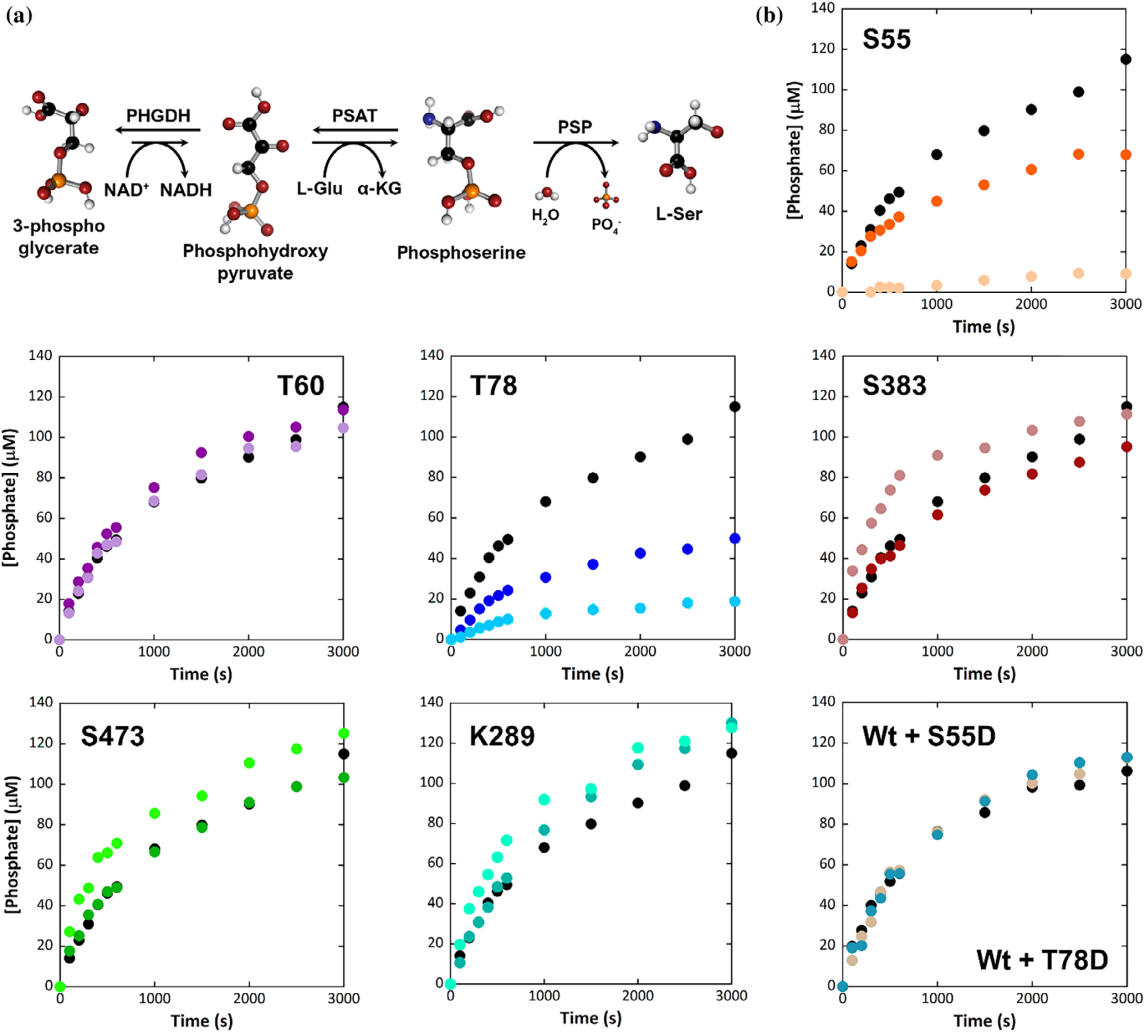
Kinetics of the in vitro reconstituted PP. (a) Reaction scheme of the phosphorylated pathway: PHGDH catalyzes the NAD‐dependent oxidation of D‐3‐phosphoglycerate into 3‐phosphohydroxypyruvate; PSAT catalyzes the transamination of 3‐phosphohydroxypyruvate to L‐3‐phosphoserine; PSP catalyzes the irreversible hydrolysis of L‐3‐phosphoserine to L‐serine. (b) Kinetics of the reconstructed PP using each PHGDH variant was followed as phosphate production by the malachite green assay. In each panel is reported the comparison of the kinetics of the wild‐type PP (wild‐type [black]) with the alanine and aspartate substitutions for each variant related to phosphorylation (S55A [orange], S55D [pale orange], T60A [dark purple], T60D [lavender], T78A [blue], T78D [light blue], S383A [firebrick], S383D [salmon], S473A [dark green], S473D [light green]) or the glutamine and arginine substitutions for variants related to acetylation (K289Q [deep teal], K289R [aquamarine]). Bottom‐right panel: Kinetics of the reconstructed PP obtained mixing an equimolar concentration of S55D or T78D and wild‐type PHGDH (0.41 μM wild‐type [black], 0.41 μM wild‐type [medium teal] + 0.41 μM S55D [beige], 0.41 μM wild‐type + 0.41 μM T78D [teal]) compared to the same amount of wild‐type enzyme alone.

The activity of the reconstructed PP was evaluated in the physiological direction by a discontinuous assay based on the quantification of the green complex formed between Malachite green reagent and the orthophosphate released during the hydrolysis of the PS into L‐Ser (Rabattoni et al., 2023). Interestingly, the trend of activity when the PHGDH variants were coupled with PSAT and PSP resembled the one recorded for the kinetics of the PHGDH variants alone (Figure 5). Particularly, the S55D and T78D substitutions resulted in a very low phosphate production (<15%, compared to the wild‐type PHGDH), suggesting that the in vitro pathway reassembly did not overcome the decrease in PHGDH activity, which is known as the rate limiting step in the PP (Rabattoni et al., 2023). Interestingly, the reconstituted PP containing an equimolar mixture of wild‐type PHGDH (0.41 μM) and either the S55D or T78D variants (0.41 μM) displayed phosphate production levels similar to those observed for a complex assembled with 0.41 μM wild‐type PHGDH alone. This indicates that the low‐activity variants do not compromise the catalytic competence of the wild‐type enzyme and have no detrimental effect on the reassembled PP incorporating wild‐type PHGDH. Notably, a faster phosphate production within 600 s of reaction is evident for the S383D, S473D, and K289Q variants (64%, 44%, and 45%, respectively) compared to the wild‐type PHGDH, a result in good agreement with their higher k_cat_ value (Table 2).

Malate is known as an alternative substrate for PHGDH (Murtas et al., 2021; Murtas et al., 2020) and has been proposed to support nuclear NADH production following nuclear translocation of the enzyme phosphorylated at S55 and S371 (Ma et al., 2021). In particular, phosphorylation at S55 was reported to enhance the malate oxidation into oxaloacetate, accompanied by NAD^+^ consumption. Under our assay conditions, the catalytic activity of the S55D variant and wild‐type PHGDH toward D,L‐malate (20 mM) was comparable (0.005–0.006 U/mg protein), corresponding to <1% of the maximal activity of the wild‐type enzyme on 3PG and 12.5% of the maximal activity reached by the S55D variant on 3PG.

### 2.6 NAD ^+^/NADH binding to the PHGDH variants

The fluorescence spectra of wild‐type PHGDH, upon excitation at 280 nm, exhibit a peak at 330 nm. The protein fluorescence intensity is quenched by adding NAD^+^ or NADH, suggesting that cofactor binding promotes conformational alterations (Murtas et al., 2021; Murtas et al., 2020). All the PHGDH variants display a lower fluorescence intensity variation following NAD^+^ or NADH binding (ΔF < 340) compared to the wild‐type PHGDH (ΔF ≈ 350, Table 3 and Figure S6), suggesting a different exposure of aromatic residues.

**TABLE 3:** Binding of NAD^+^ and NADH to wild‐type and variants of PHGDH.

Variant	Wild‐type	S55A	S55D	T60A	T60D	T78A	T78D	S383A	S383D	S473A	S473D	K289Q	K289R
**K_d_ (μM) NAD^+^**													
First phase	81.8 ± 3.9	41.7 ± 4.2	13.3 ± 2.6	86.4 ± 10.1	91.4 ± 8.1	49.3 ± 3.3	71.1 ± 11.5	1.2 ± 0.2	1.2 ± 0.2	183 ± 19	1.4 ± 0.2	157 ± 21	127 ± 13
Second phase	—	—	160 ± 10	—	—	—	—	71.1 ± 5.6	26.5 ± 1.0	—	80.6 ± 8.8	—	—
**Fluorescence variation**													
Total	345	250	260	245	300	310	285	230	320	220	280	250	380
First phase (%)	100	100	45	100	100	100	100	39	50	100	45	100	100
Second phase (%)	—	—	54	—	—	—	—	61	50	—	55	—	—
**K_d_ (μM) NADH**													
First phase	0.3 ± 0.1	0.5 ± 0.1	1.7 ± 0.4	1.5 ± 0.3	0.8 ± 0.2	0.2 ± 0.1	0.7 ± 0.1	0.2 ± 0.1	0.2 ± 0.1	21.7 ± 3.9	0.1 ± 0.1	0.3 ± 0.1	1.0 ± 0.1
Second phase	28.8 ± 7.7	21.7 ± 2.5	24.1 ± 1.8	29.8 ± 4.3	21.3 ± 1.8	15.7 ± 1.8	17.5 ± 1.1	19.3 ± 1.5	20.2 ± 1.7	—	20.5 ± 2.7	52.6 ± 03.5	46.1 ± 3.9
**Fluorescence variation**													
Total	355	290	270	290	320	300	325	250	340	250	275	260	395
First phase (%)	28	34	48	55	43	36	33	36	32	100	29	27	39
Second phase (%)	72	66	52	45	57	64	67	64	67	—	71	73	61

*Note*: Measurements were carried out at 15°C, in 10 mM potassium phosphate buffer, pH 7.0; K_d_ values are mean ± SEM (*n* = 2).

The NADH binding for PHGDH wild‐type and acetylation and phosphorylation mimetic variants is a biphasic process, with a first phase characterized by a low K_d_ (between 0.3 and 1 μM) and a second one showing a higher K_d_ value (around 50‐fold higher than the first one), also associated with the largest change in the fluorescence intensity (between 60% and 72%) (Table 3).

The NAD^+^ binding to wild‐type PHGDH is a monophasic process, with a K_d_ of 82 μM, similar to that observed for S55A, T60A, T60D, T78A, T78D, S473A, K289Q, and K289R variants. Compared to the wild‐type PHGDH, the S55A and T78A PHGDH variants have a lower K_d_ value (42 and 13 μM, respectively), whereas K289Q, K289R, and S473A PHGDH variants show K_d_ values >120 μM, see Table 3. On the contrary, the S55D, S383A, S383D, and S473D variants display a biphasic binding process (Figure S7) with a very low K_d_ for the first phase (between 1 and 13 μM) and a higher figure for the second phase (between 20 and 160 μM).

Altogether, the NAD^+^/NADH binding to PHGDH is only slightly altered by the introduced substitutions.

### 2.7 Subcellular localization of the PHGDH variants

In light of recent findings (Ma et al., 2021) which demonstrated that the first enzyme of the L‐serine biosynthesis pathway can acquire a nuclear role under nutrient stress through the importin ẞKPNB1, specifically following phosphorylation of residue S371 by p38 kinase, we sought to investigate whether the newly identified PTMs at the aforementioned residues might similarly promote nuclear translocation of PHGDH in non‐tumor cells. Two nuclear translocation sequences have been proposed: NLSa, residues L6‐K33; NLSb, V31‐T60 (Ma et al., 2021). Bioinformatic analyses using online available tools, for example, cNLS Mapper (https://nls-mapper.iab.keio.ac.jp/cgi-bin/NLS_Mapper_form.cgi) and NucPred (https://nucpred.bioinfo.se/cgi-bin/single.cgi), failed in identifying any canonical nuclear localization sequences (NLS) in either wild‐type PHGDH or its variants mimicking phosphorylation or acetylation (Figure S8). Even the S371D variant, included as a putative positive control, yielded a negative prediction. Interestingly, PSORT II (https://psort.hgc.jp/form2.html) predicted a slightly higher nuclear localization for the S55D and T60A variants.

**FIGURE 6:**
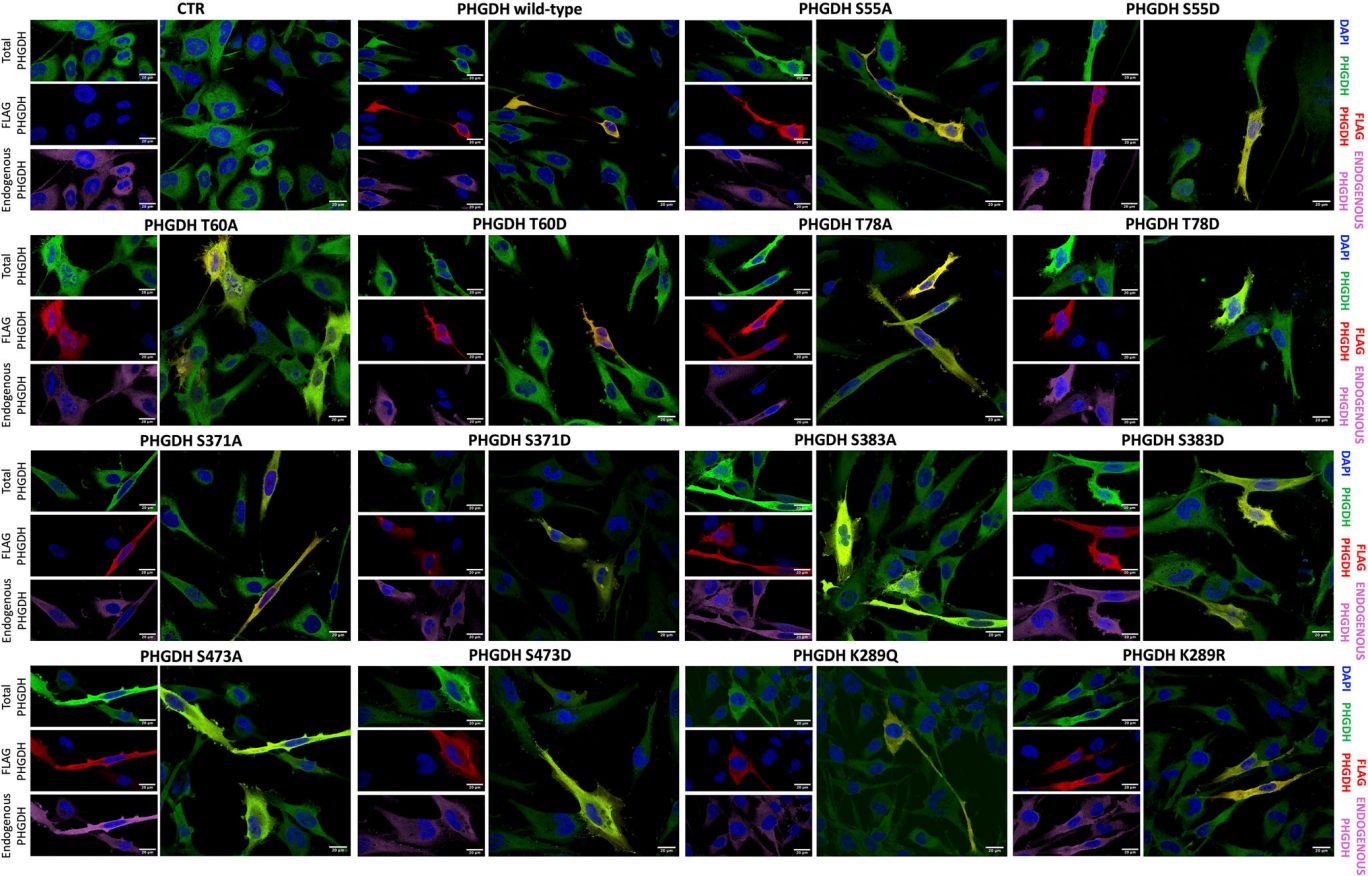
Confocal analysis of U251 cells ectopically expressing PHGDH wild‐type and variants to assess subcellular distribution. Localization of recombinant PHGDH (detected with an anti‐FLAG antibody, red) was evaluated relative to total PHGDH (detected with an anti‐PHGDH antibody, green). Because the anti‐PHGDH antibody recognizes both ectopically and endogenously expressed PHGDH, endogenous PHGDH (shown in pink) was generated by digitally subtracting the FLAG signal from the total PHGDH signal. Nuclei were visualized with DAPI (blue). Image acquisition parameters were standardized using untransfected control cells: the 405 nm laser was optimized for DAPI, the 488 nm laser for Alexa Fluor 488 (total PHGDH), and the 561 nm laser for Alexa Fluor 546 (FLAG‐tagged PHGDH). The 561 nm settings were carefully adjusted to eliminate non‐specific signal. Scale bar: 20 μm.

To deepen insight into the subcellular localization of PHGDH, we transiently transfected U251 glioblastoma cells, which endogenously express the PP's enzymes (Murtas et al., 2023), with the pcDNA3.1+/C‐(K)‐DYK‐hPHGDH‐1xFLAG encoding the wild‐type enzyme or the variants fused to a C‐terminal FLAG epitope, and explored their distribution by imaging immunostained cells fixed 48 h post‐transfection at the confocal microscope. Immunofluorescence analyses revealed no major differences in the distribution of the variants compared to the wild‐type, with all forms being predominantly cytosolic. Notably, the S371D PHGDH exhibited consistent accumulation in small perinuclear aggregates, likely vesicular structures (Figure 6), suggesting a response related to nuclear signaling, albeit insufficient for nuclear entry under these conditions and/or in this cellular system, and lower levels were observed for both K289Q and K289R variants.

Post‐imaging analyses involved quantifying the fluorescence intensity of recombinant proteins within nuclei and whole cells, followed by estimating their three‐dimensional distribution by assuming spherical geometry and calculating volumes from ROI‐derived radii. Across 628 cells, no significant differences in nuclear localization were observed between wild‐type PHGDH and variants, in line with bioinformatic predictions (Figure 6, Table S5). The sole exception was the T60A variant, which exhibited a modest but statistically significant increase in nuclear signal (mean nuclear fraction: wild‐type = 6.9%, T60A = 11.1%).

**FIGURE 7:**
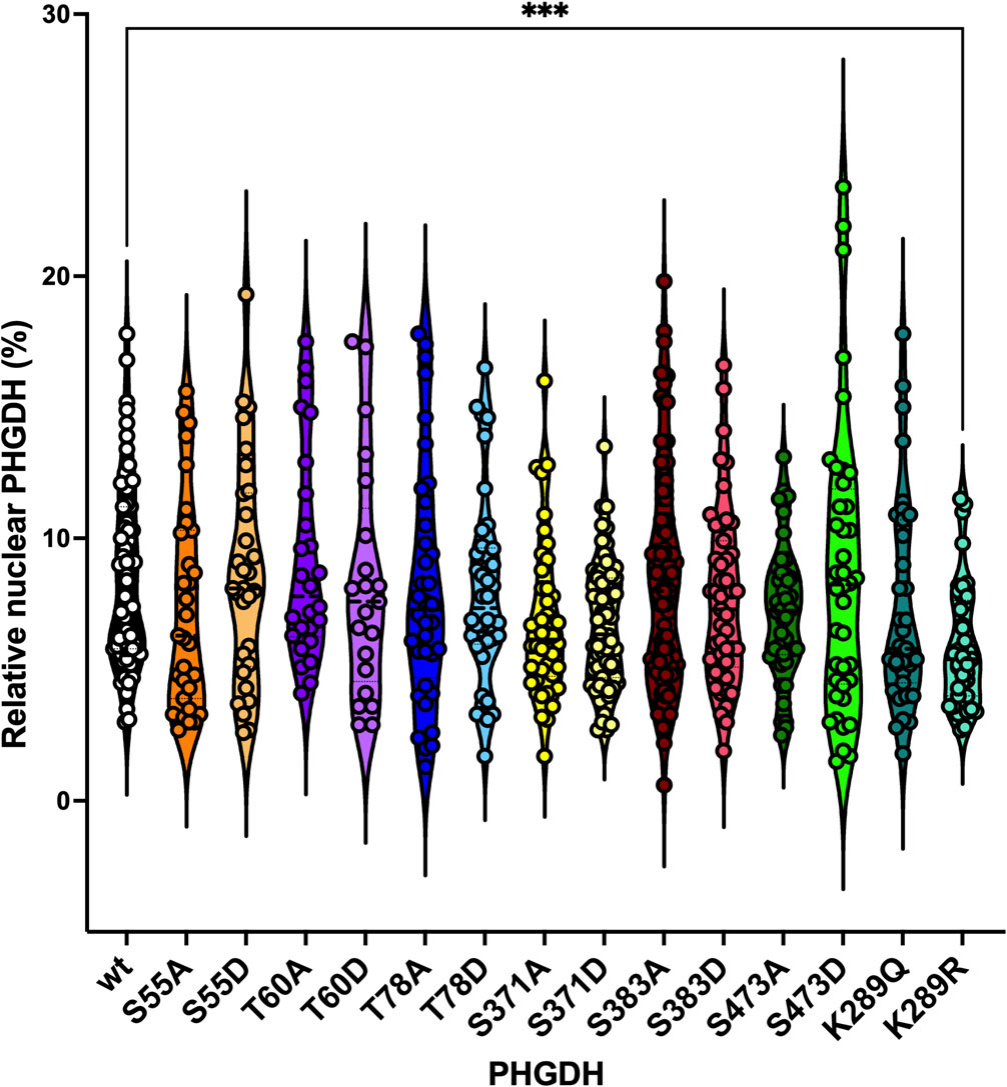
Violin plot illustrating the nuclear fraction of recombinant PHGDH wild‐type and variants after normalization on expression levels. The nuclear fraction was calculated as the ratio of nuclear to total cellular fluorescence signal for each individual cell after the normalization procedure. The analysis includes the following groups: Wild‐type (black, *n* = 59), S55A (orange, *n* = 31), S55D (pale orange, *n* = 30), T60A (dark purple, *n* = 26), T60D (lavender, *n* = 21), T78A (blue, *n* = 36), T78D (light blue, *n* = 38), S371A (yellow, *n* = 43), S371D (pale yellow, *n* = 61), S383A (firebrick, *n* = 73), S383D (salmon, *n* = 49), S473A (dark green, *n* = 43), S473D (light green, *n* = 40), K289Q (deep teal, *n* = 35), K289R (aquamarine, *n* = 43).

To account for variability in ectopic protein expression and considering the markedly lower levels observed for the recombinant K289Q and K289R variants, we repeated the analysis after having normalized nuclear and total signals to their respective mean values (Table S6). This correction revealed that the apparent increase in nuclear localization for T60A PHGDH was likely an artifact of its higher expression level (Table S6), as indicated by Mean Gray Value, an ImageJ's parameter often used for quantifying signals (Figure 7). Interestingly, normalization suggested a reduced nuclear localization for K289R and S371D PHGDH relative to the wild‐type protein, even though the latter narrowly failed to reach statistical significance. While the functional implications of K289R warrant further study, the nuclear exclusion of S371D PHGDH is consistent with its observed perinuclear aggregation.

To investigate whether the acetylation of K289 affects PHGDH protein stability, U251 cells ectopically expressing either wild‐type PHGDH, the acetylation‐mimic K289Q variant, or the non‐acetylatable K289R variant were subjected to cycloheximide (CHX) chase analysis. As shown in Figure S9, both the wild‐type and K289R variants displayed slow decay kinetics, retaining ~70%–75% of their initial protein levels after 30 h of CHX treatment, consistent with PHGDH being an intrinsically long‐lived and stable enzyme. In contrast, the K289Q variant did not exhibit a comparable decrease in abundance under the same conditions, suggesting that acetylation‐mimic substitution at this position alters PHGDH reducing the turnover dynamics. Control cells treated with DMSO in place of CHX maintained constant protein levels throughout the assay (data not shown), confirming that the observed decrease reflects inhibition of protein synthesis rather than experimental variability.

## 3 DISCUSSION

The “phosphorylated pathway” is the primary source of L‐Ser in the adult brain, since this amino acid—in spite of being freely available in the blood arising from dietary intake, enzymatic conversion of glycine, and protein and phospholipid degradation—diffuses very poorly across the blood–brain barrier (Maugard et al., 2021). Importantly, the PP supplies the glutamatergic neurotransmitter system with L‐Ser that is the precursor of two endogenous co‐agonists, D‐Ser and Gly, which finely regulate the activation state of NMDARs (Mothet et al., 2015). PHGDH, the first enzyme of the PP, is preferentially expressed in astrocytes (Furuya, 2008); L‐Ser is, then, either converted to Gly through the enzymatic action of serine hydroxymethyltransferase (SHMT, EC 2.1.2.1) or released and taken up by neurons, which express serine racemase (SR, EC 5.1.1.18) that catalyzes its racemization into D‐Ser (Takagi et al., 2022). Therefore, as further confirmed by the early embryonic lethality observed in Phgdh^−/−^ homozygous knock‐out mice (Yoshida et al., 2004), this short cytosolic pathway is crucial to guarantee the correct brain functioning. In line with these findings, we recently reported alterations in L‐Ser biosynthesis due to increased levels of PHGDH, PSAT (as well as SR) in the hippocampus of AD patients (Maffioli et al., 2022). Intriguingly, we showed that the L‐Ser and D‐Ser metabolisms are differently affected (and possibly differently regulated) depending on the individual sex (Maffioli et al., 2022). Furthermore, the discovery of a putative metabolon organization of the PP enzymes (the “serinosome”) (Rabattoni et al., 2023) might imply a sophisticated regulatory strategy of the PP depending on the activity and stability of this transient complex.

In the present work, IP samples from post‐mortem hippocampal tissue lysates of AD and healthy subjects were subjected to MS analyses to identify PTMs, focusing on phosphorylation and acetylation. In this regard, numerous studies have already reported PTMs in PHGDH, but exploring them in the oncogenic context only, that is, in tumors and cancer cell lines (Ma et al., 2013; Ma et al., 2021). In this context, in addition to phosphorylation, PHGDH was reported to be acetylated (Wang et al., 2020), methylated (Wang et al., 2023; Yamamoto et al., 2024), ubiquitinated (Liu et al., 2020a; Liu et al., 2020b), and (in vitro) nitrosylated (Bruegger et al., 2018). A list of the kinases identified or predicted as responsible for phosphorylation at each position is reported in Table 1. PHGDH phosphorylation of S55, T57, and T78 by the tumor suppressor PKCζ has been reported to inhibit enzymatic activity preventing cells from acquiring the necessary plasticity to adapt their metabolism to the use of glutamine, which can originate from the PP, in the absence of glucose (Ma et al., 2013). Furthermore, under nutrient stress, p38 mitogen‐activated protein kinase (p38 MAPK) phosphorylates PHGDH at S371 causing its translocation to the nucleus, where the phosphorylation by AMPK at S55 triggers the increase of a non‐canonical malate oxidation into oxaloacetate by the modified PHGDH that concomitantly consumes NAD^+^. The reduced NAD^+^ levels, in turn, repress the transcription factor c‐Jun, whose activity is linked to cell growth inhibition, thus sustaining tumor progression (Ma et al., 2021). On the other hand, Wang and colleagues revealed that the ubiquitination of PHGDH by the RING finger E3 ubiquitin ligase (RNF5) and the reversible glucose‐dependent acetylation at K58 mediated by the acetyltransferase Tip60 and the deacetylase SIRT2, regulate the PP's enzyme levels by affecting its cellular stability. Glucose deprivation decreases PHGDH acetylation promoting the interaction of the enzyme with RNF5, thus leading to its degradation that, eventually, results in L‐Ser, D‐Ser, and Gly decrease, reactive oxygen species increase and cell growth inhibition (Wang et al., 2020). Here, MS analyses did not detect any different phosphorylation pattern among the four groups (i.e., males/females, healthy/AD) suggesting that, unlikely, this PTM might not contribute to the sex‐dependent AD onset and development. Complementary quantitative MS approaches, which would provide insight into site occupancy and the relative extent of phosphorylation at each position, could not be applied due to the very limited amount of material available. On the contrary, acetylation of PHGDH K289 residue was found in all categories of subject, with the exception of AD‐affected males.

Our study identified the PHGDH residues that are typically phosphorylated in the human hippocampus, at least during aging; among them, S383 had not previously been observed—and only weakly predicted—to undergo modification. Notably, all the identified residues (S55, T60, T78, S93, and S473) are highly conserved among species and not related to pathological single‐nucleotide polymorphisms (SNPs). Studies on phosphomimetic PHGDH variants show that only phosphorylation at residues S55 and T78 (the residues modified by PKCζ) (Ma et al., 2013) strongly affects the enzymatic activity (and thus the overall PP‐mediated L‐Ser synthesis), as well as the protein solubility (see below). S55 and T78 are in close proximity to 3PG; thus, phosphorylation should result in an electrostatic repulsion of the substrate, this affecting the kinetic properties (Figure 2c). The full‐length tetrameric structure of PHGDH was recently modeled using AlphaFold combined with molecular dynamics refinement (Riva et al., 2024). In this model, tetramerization is primarily mediated by the C‐terminal ACT domain, with key interface residues including R454, P475, L478, P479, L482, L492, and Y495, which engage in a network of pairwise non‐covalent interactions (see Figure S10). Substitution at position S473 is predicted to perturb this interface, potentially altering local packing and promoting exposure or reinforcement of aggregation‐prone regions within the ACT domain at the dimer–dimer contact surface. This structural rearrangement could explain the pronounced aggregation propensity of the S473D/A variants. Even S55D, S383A, and K289Q/R are prone to aggregation at all tested protein concentrations (Figure S10). Previous computational analyses of binding energy changes upon phosphorylation have suggested that protein–protein interactions are frequently not significantly perturbed by this modification (Nishi et al., 2011). In line with this notion, kinetic assays of L‐Ser synthesis within the reconstituted phosphorylated pathway showed that none of the phospho‐ or acetyl‐mimetic PHGDH variants exhibited impaired complex formation. Instead, the observed changes in L‐Ser production rates were directly attributable to altered catalytic properties of the individual PHGDH variants, thereby confirming the rate‐limiting role of PHGDH within this metabolic pathway.

Notably, both phosphorylation and acetylation do not seem to affect the cofactor binding and to favor the nuclear localization of PHGDH (all modified residues do not belong to the 110–160 region recently proposed to interact with DNA) (Wang et al., 2025). Our results suggest that the phosphorylation of S55 is not sufficient for nuclear localization of PHGDH, thus better explaining the interconnection with S371 phosphorylation observed in tumor cells (Ma et al., 2021). This discrepancy with previous findings (Ma et al., 2021) may be attributable to the absence of additional stimuli beyond the simple phosphomimetic substitution. Concerning the previously reported increased activity on malate of PHGDH phosphorylated at S55, our results do not confirm such evidence: the activity of the S55D variant on malate is lower than that of the wild‐type PHGDH and strongly lower than that on the physiological substrate 3PG. In addition, this study highlights significant differences in subcellular targeting of the phosphorylation‐mimicking S55D variant compared to cancer cells, probably reflecting the different physiological levels and the metabolomic alterations in tumor development (especially the need to maintain high NADH levels in the nucleus).

Regarding K289 acetylation—a modification previously reported to be absent in tumor cells (Ma et al., 2013)—our data suggest that it alters the secondary structure of PHGDH and promotes aggregate formation, while leaving 3PG dehydrogenase activity, L‐Ser production efficiency, and NADH interaction largely unaffected. The increased cellular stability observed for the K289Q variant, compared with both the wild‐type protein and the non‐acetylatable K289R one, supports the notion that acetylation at this position has a stabilizing effect on PHGDH turnover. No evidence of altered nuclear localization was detected.

A correlation analysis was performed to relate SEC‐derived tetramer abundance to four independent biochemical parameters—thermal stability (T_m_), catalytic efficiency (k_cat_/K_M_), α‐helix content, and total secondary‐structure content (α‐helix + β‐sheet)—at each protein concentration. All statistical analyses employed Spearman's rank correlation coefficient, which does not assume linearity and is well‐suited for identifying monotonic trends in datasets with limited sample size and potential nonlinear behavior. Notably, SEC analyses revealed two distinct aggregation behaviors among PHGDH variants. One subset of variants exhibited substantial tetramer loss already at low protein concentrations, indicative of intrinsic defects in quaternary assembly. In contrast, other variants maintained relatively stable tetrameric assembly at low concentration but showed pronounced, concentration‐dependent tetramer loss. Across all concentrations tested, both baseline tetramer abundance and concentration‐dependent tetramer loss displayed only weak monotonic associations with thermal stability, secondary‐structure content, and catalytic efficiency (Figure S11). With respect to enzymatic turnover, a significant negative association between baseline tetramer abundance and k_cat_ was observed at 0.33 mg/mL (ρ = −0.68; Figure S11A); this correlation progressively weakened at higher protein concentrations and was absent at 10 mg/mL. This trend reflects the observation that several catalytically impaired variants retain their quaternary structure, particularly when substitutions introduce charged residues into the active site. Taken together, PTM‐mimetic substitutions affect quaternary assembly and concentration‐dependent oligomerization while largely preserving global fold stability. Consequently, strong catalytic impairments are not systematically associated with increased aggregation, as exemplified by the S55D and T78D variants, which introduce a negative charge in close proximity to the substrate 3‐PG.

The main limitation of this study is the inability to determine the relative stoichiometry of the individual modifications—as well as their combinatorial occurrence—due to the low abundance of PHGDH in the human hippocampus. This currently prevents a comprehensive evaluation of how multiple phosphorylation events and PTM crosstalk cooperatively modulate PHGDH conformation and activity within the serinosome. Future quantitative and top‐down proteomic strategies will be crucial to resolve site occupancy and combinatorial PTM patterns.

Altogether, the investigation of PTMs in human hippocampus samples adds a new layer of complexity to the regulation of serine biosynthesis in the brain and its disruption in neurodegenerative diseases, unveiling novel mechanistic insights, and potential therapeutic targets for AD and related disorders.

## 4 MATERIALS AND METHODS

### 4.1 Patient cohorts and brain samples

Control (CTR) and Alzheimer's disease (AD) brain samples (hippocampus) (Table S1) were obtained from the Medical Research Council (MRC) London Neurodegenerative Diseases Brain Bank hosted at the Institute of Psychiatry, Psychology and Neuroscience, KCL, and were previously used in (Maffioli et al., 2022). All cases were collected under informed consent and the bank operates under a license from the Human Tissue Authority and ethical approval as a research tissue bank (08/MRE09/38 + 5). Neuropathological evaluation for neurodegenerative diseases was performed in accordance with standard criteria. All methods were carried out in accordance with relevant guidelines and regulations and the study was approved by the Institutional Review Board of the University of Rome “Tor Vergata” (Protocol No. 98.18). All sampled individuals were European; men and women showed a comparable age of death (Mann–Whitney test >0.5). The age distribution of CTR samples was in the 81.2 ± 4.9 range (mean ± SD) with PMI intervals included between 3 and 48 h. Similar age distribution was obtained for AD samples (82.8 ± 5.9), with PMI intervals included between 4 and 20.5 h. Considering sex separation: CTR females: *n* = 8, age distribution = 81.2 ± 5.25, 3 < PMI < 35 h; CTR males: *n* = 6, age distribution 81.3 ± 4.8, 6 < PMI < 48 h; AD females: *n* = 8, age distribution 85.1 ± 4.5, 4 < PMI < 13; AD males: *n* = 5, age distribution 80.0 ± 6.45, 5.25 < PMI < 20.5. CTRs were defined as having no evidence of dementia, that is, donors with minimal aging alterations, mild cognitive impairment, and neuropathological findings insufficient to meet criteria for AD. All patients with AD had Braak stages in the range of IV–VI. Tissue samples of 500–1000 mg were dissected and stored at −80°C until analysis.

### 4.2 Preparation of brain tissue samples for immunoprecipitation

Hippocampal tissue samples (10 mg) from healthy and AD‐affected subjects were homogenized (100 μL) in 20 mM Hepes, pH 8.0, 1 mM EGTA, 5% glycerol, 0.4% NP‐40, added of 1X complete mini protease (Roche), 1X phosphatase inhibitor cocktail (Cell Signaling Technology), using a pellet micro‐pestle and then subjected to sonication (3 cycles, 20 and 30 s in ice, each). After 10 min of incubation in ice, samples were centrifuged for 10 min at ~16,000 g at 4°C. The clarified supernatants were pooled (maintaining separate sample groups: healthy individuals and AD patients, males and females) to obtain a sufficient amount of starting material for the immunoprecipitation (IP), which was performed in parallel on at least four identical technical replicates. Total protein concentration of pooled samples was quantified using the Bradford protein assay (Bio‐Rad).

### 4.3 PHGDH immunoprecipitation

Individual hippocampal samples were previously evaluated for the endogenous amount of PHGDH by Western blot analyses (Table S1) (Maffioli et al., 2022). An optimized protocol was set up for immuno‐precipitating PHGDH: 250 μg of total proteins (1.25 μg/μL, total volume 200 μL) were precleared by adding 50 μL of Dynabeads protein G (Invitrogen) for 60 min at 4°C with constant rotation. The precleared supernatant was added with 1.5 μg of rabbit polyclonal anti‐PHGDH antibody (HPA024031, Sigma) and incubated overnight at 4°C with constant rotation. In parallel, to assess the isolation of endogenous PHGDH, a positive control, hippocampal lysate added with 100 ng of recombinant PHGDH and incubated with 1.5 μg of rabbit polyclonal anti‐PHGDH antibody (HPA024031, Sigma), and a negative control, hippocampal lysate added with 100 ng of recombinant PHGDH and incubated with 1.5 μg of a rabbit polyclonal anti‐hDAAO (Davids Biotechnologie, Sacchi et al., 2008) (unrelated) antibody, were included. Samples were added with 50 μL of Dynabeads protein G and incubated for 60 min at room temperature with constant rotation to isolate the immune‐complexes. The immunoprecipitated protein was recovered after extensive washes in PBS, 0.02% Tween‐20, performing a double elution: (a) in 50 mM glycine pH 2.8 and (b) in Sample buffer 1X (SB1X) for blotting analysis; alternatively, beads were flash frozen in liquid nitrogen and stored at −80°C for subsequent MS analyses.

To evaluate the yield of the IP experiments, samples before (PRE‐IP sample) and after the incubation with the antibody and beads (POST‐IP sample) were compared, also evaluating protein eluted with glycine (glycine‐eluted IP sample) or SB1X (SB1X‐eluted IP sample). Protein samples were loaded onto a 12% SDS‐PAGE gel along with a molecular weight marker (PageRuler Prestained Protein Ladder, Thermo Scientific) and mixtures containing fixed amounts of the recombinant protein (10–30 ng for PHGDH). After gel electrophoresis, proteins were transferred to a PVDF membrane (Millipore) via electroblotting. The membrane was incubated with blocking solution (Tris‐buffered saline (TBS) 0.1% Tween‐20, 4% non‐fat dry milk) for 2 h at room temperature, then with primary antibodies appropriately diluted in TBS 0.05% Tween‐20, 2% non‐fat dry milk and, after extensive washes, with secondary antibodies diluted in TBS 0.05% Tween‐20 (Table S7).

Chemiluminescent signals corresponding to the proteins of interest were detected and analyzed by the Odyssey Fc apparatus equipped with the ImageStudio software (LI‐COR Biosciences). IP yield was estimated by comparing the band intensity of the target protein in the post‐IP sample with that in the pre‐IP sample; the identity of the recovered protein was confirmed by comparing the bands observed in the glycine‐eluted and SB1X‐eluted IP samples with that corresponding to recombinant PHGDH.

### 4.4 Preparation of immunoprecipitated samples for MS

Since preliminary mass spectrometry analyses revealed that endogenous PHGDH remained largely bound to the beads upon glycine elution, both the bead‐bound and glycine‐eluted fractions from each sample were analyzed separately, and the resulting data were combined to obtain the final quantitative assessment. In detail, (i) beads were digested with 5 μg/mL sequence grade trypsin in 2 M urea, 50 mM Tris HCl pH 7.5, for 30 min at 27°C in agitation (~800 rpm). Upon centrifugation and collection of the supernatant (SN1), the beads were washed twice with 2 M urea, 50 mM Tris HCl, 1 mM DTT, centrifuged for 30 s at 7000 rpm and the supernatant was collected (SN2). The two supernatants (SN1 and SN2) were pooled and incubated with 2 M urea, 50 mM Tris HCl pH 7.5 and 5 μg/mL sequence grade trypsin overnight at 37°C (Zambonelli et al., 2003). Samples were incubated with 5 mg/mL iodoacetamide (IAA) for 30 min in the dark. The material was then treated with trifluoroacetic acid (TFA) to stop the digestion and desalted in Zip‐Tip C18 before MS analysis; (ii) glycine‐eluted fractions were reduced with 1 mM DTT for 30 min at 56°C and alkylated with 20 mM IAA for 30 min at room temperature. The protein sample was digested with 5 μg/mL sequence grade trypsin for 16 h at 37°C (protein: enzyme ratio of 10:1). The collected peptides were desalted using Zip‐Tip C18 before MS analysis (Capraro et al., 2013).

### 4.5 nLC‐MS/MS analysis

Three biological replicates for each of the four groups (female and male AD patients and female and male healthy subjects) were processed for the LC‐ESI MS/MS. The samples were analyzed in two steps. First, each sample was analyzed by an untargeted approach. NanoHPLC coupled to MS/MS analysis was performed on Dionex UltiMate 3000 directly connected to an Orbitrap Fusion Tribrid mass spectrometer (Thermo Fisher Scientific) by a nano‐electrospray ion source. Peptide mixtures were enriched on 75 mm ID 3150 mm EASY‐Spray PepMap RSLC C18 column (Thermo Fisher Scientific) and separated using the LC gradient: 1% acetonitrile (ACN) in 0.1% formic acid for 10 min, 1%–4% ACN in 0.1% formic acid for 6 min, 4%–30% ACN in 0.1% formic acid for 147 min and 30%–50% ACN in 0.1% formic for 3 min at a flow rate of 0.3 mL/min. Orbitrap‐MS spectra of eluting peptides were collected over a m/z range of 375–1500 at resolution of 120,000, operating in a data‐dependent mode with a cycle time of 3 s between master scans. HCD MS/MS spectra were acquired in Orbitrap at resolution of 15,000 using a normalized collision energy of 35%, and an isolation window of 1.6 m/z. Dynamic exclusion was set to 60 s. Rejection of +1, and unassigned charge states were enabled. Raw label‐free MS/MS files from Thermo Xcalibur software (version 4.1) were analyzed using Proteome Discoverer software (version 1.4, Thermo Fisher Scientific) and searched with the Sequest algorithm against the human PHGDH from UniProt 17‐11‐2021. Only peptides with medium confidence and a high cross‐correlation score (≥1.5) were considered. The minimum required peptide length was set to 6 amino acids with carbamidomethylation as a fixed modification, Ser/Thr/Tyr phosphorylation, Lys acetylation, Met oxidation, and Arg/Gln deamidation as variable modifications (Nonnis et al., 2021; Schulte et al., 2016). Then, a targeted analysis was carried out on an aliquot of each sample to confirm the modified residues identified by the untargeted analysis. Parallel reaction monitoring (PRM) was carried out using a Dionex UltiMate 3000 nanoHPLC coupled with a Q‐Exactive Orbitrap mass spectrometer (Thermo Fisher Scientific, San Jose, CA, USA). Peptides were separated on 75 mm ID 2150 mm EASY‐Spray PepMap RSLC C18 column (Thermo Fisher Scientific). A predefined inclusion list was set up with LC tandem mass spectrometry (LC‐MS/MS) parameters. In PRM, a full mass spectrum at 70,000 resolution relative to m/z 200 was followed by PRM scans at 35000 resolution (AGC target 2 × 105, 100 ms maximum injection time). Ion activation/dissociation was performed at a normalized collision energy of 30% in a higher‐energy c‐trap dissociation (HCD) collision cell. Mass spectrometry data files were analyzed by Skyline software (version 22.2).

### 4.6 Expression in *Escherichia coli* and purification of PHGDH variants

Mutagenesis reactions were performed on the pETM11‐His‐hPHGDH expression plasmid (Murtas et al., 2021; Murtas, Marcone, Sacchi, & Pollegioni, 2020; Murtas et al., 2024) using the primers reported in Table S8. The conditions resulting in the highest production yield for the wild‐type PHGDH were used to optimize the expression of each variant: transformed *Escherichia coli* BL21(DE3) LOBSTR cells were grown until an OD_600nm_ value of 0.6 at 37°C was reached, then 0.1 mM isopropyl β‐D‐1‐thiogalactopyranoside (IPTG) was added and cells were incubated at 17°C for 20 h. Cells were harvested by centrifugation (8000 *g*, 10 min, 4°C) and stored at −20°C. The His‐tagged PHGDH variants were purified by HiTrap chelating chromatography (Amersham Biosciences, Amersham, UK) as reported for the wild‐type enzyme (Murtas et al., 2021; Murtaset al., 2020). The final enzyme preparations were stored at −80°C in 50 mM HEPES, pH 7.0, 5% (vol/vol) glycerol. The enzyme concentration was determined spectrophotometrically by using the theoretical extinction coefficient at 280 nm (40.45 mM^−1^ cm^−1^).

### 4.7 Activity assay and kinetic measurements

The PHGDH activity in the forward direction was determined by a chemical coupled assay using hydrazine monohydrate (Sigma, ID: 225819) (Riva et al., 2024). The reaction was followed at 340 nm by monitoring the time course of NADH production at 37°C in 1 cm quartz cuvette with a Jasco V‐750 spectrophotometer (Jasco Co., Cremella, Italy). The assay was performed under standard conditions: 0.1 μM PHGDH (2.39 μg), 2.5 mM 3PG, 1.5 mM NAD^+^, and 200 mM hydrazine monohydrate in 25 mM 4‐(2‐hydroxyethyl)‐ 1‐piperazineethanesulfonic acid (HEPES) buffer, pH 7.0. The latter assay was used to determine the apparent kinetic parameters according to a classical Michaelis–Menten equation.

The activity of the reconstructed PP was evaluated by monitoring the free phosphate production by a discontinuous assay using Malachite‐green reagent (Sigma, MAK308) (Marchesani et al., 2021). The assay was performed at physiological concentrations of the three PP's enzymes, that is, 0.82 μM PHGDH, 1.14 μM PSAT, and 0.12 μM PSP (Maffioli et al., 2022).

### 4.8 Determination of the oligomerization state

The oligomerization state was investigated by size‐exclusion chromatography using a Superdex 200 Increase 10/300 GL column (Cytiva, Milano, Italy; 1–600 kDa separation range) on an AKTA system. The column was equilibrated in 50 mM HEPES, pH 7.0, 0.15 M NaCl, and loaded with 200 μL of PHGDH variants (at 0.33, 1, 3.33, and 10 mg/mL) after spinning the sample at 13,000 rpm for 15 min at 4°C.

### 4.9 Spectral measurements

Circular dichroism (CD) spectra were recorded using a Jasco J‐815 spectropolarimeter (Jasco Co., Cremella, Italy) in 10 mM potassium phosphate, pH 7.0, at 15°C. The cell pathlength was 1 mm for measurements in the 200 to 250 nm region (0.1 mg protein/mL) and 10 mm for measurements in the 250 to 350 nm range (1 mg protein/mL) (Caldinelli et al., 2010). Temperature ramp experiments were performed in 10 mM potassium phosphate, pH 7.0, with a temperature gradient of 0.5°C/min (20–100°C range) using a software‐driven, Peltier‐equipped CD spectropolarimeter and recording the signal at 220 nm (Caldinelli et al., 2010).

The cofactor dissociation constants (K_d_ values) were estimated by titrating 1 μM of each variant with increasing amounts of NAD^+^ or NADH and following the protein fluorescence quenching at 330 nm in 20 mM potassium phosphate, pH 7.0, at 15°C (to increase protein stability)(Murtas et al., 2024). K_d_ values were determined by fitting the data to a hyperbolic function: $\Delta F=\frac{{\Delta F}_{\max}\cdot \left[\text{ligand}\right]}{K_{d}+\left[\text{ligand}\right]}$, where Δ*F* is the recorded fluorescence intensity change and Δ*F*_max_ is the maximal fluorescence intensity change.

### 4.10 Constructs for over‐expression of PHGDH variants in U251 mammalian cells

Point mutations were introduced in pcDNA3.1+/C‐(K)‐DYK‐hPHGDH‐1xFLAG (U362CGJ270–1, GenScript Piscataway, NJ) using the Quickchange LightningTM site‐directed mutagenesis kit (Agilent Technologies, Santa Clara, CA) and the primers listed in Table S9. All constructs were verified by DNA sequencing. The U251 human glioblastoma cell line (ATCC) was cultured in Dulbecco's Modified Eagles' Medium (DMEM, Euroclone, Pero, Milan, Italy) supplemented with 10% fetal bovine serum (FBS), 1 mM sodium pyruvate, 2 mM L‐glutamine, 1% non‐essential amino acid, 1% penicillin–streptomycin, and 1% amphotericin B, at 37°C in 5% CO_2_. Cells were daily evaluated at the microscope for vitality and density: subculturing was performed when cells reached 80%–90% of confluence by treatment with EDTA trypsin.

### 4.11 Transient and stable transfection of U251 human cells

For immunolocalization studies, human U251 glioblastoma cells were seeded into 24‐well plates (0.5 × 10^5^ cells/well) on gelatinized coverslips (diameter 12 mm; Thermo Scientific, Waltham, MA). At 24 h after seeding, cells were transfected with 0.5 μg plasmid DNA (pcDNA3.1‐hPHGDH‐1xFLAG wild‐type or variants) using VibroFect technology (Vectorialis, Milan, Italy) and following the manufacturer's instructions (Sacchi et al., 2025). The cells were fixed (on coverslips) 48 h after transfection. Fixation was performed after extensive washings with phosphate‐buffered saline (PBS, 10 mM dibasic sodium phosphate, 2 mM monobasic potassium phosphate, 137 mM NaCl, 2.7 mM KCl, pH 7.4) by incubating coverslips in 4% p‐formaldehyde for 10 min at room temperature.

For protein stability studies (see below), cells were seeded into 6‐well plates (2.0 × 10^5^ cells/well). At 24 h after seeding, cells were transfected with 2.5 μg plasmid DNA (pcDNA3.1‐hPHGDH‐1xFLAG wild‐type, K289Q, or K289R variants) using VibroFect technology and following the manufacturer's instructions (Sacchi et al., 2025). To select cell clones stably expressing PHGDH‐1xFLAG proteins, transfected cells were maintained in the previously described culture medium added with 0.4 μg/mL G418 for ~3 weeks. The isolated clones were verified for exogenous‐flagged PHGDH protein expression by Western blot analysis using a polyclonal rabbit anti‐FLAG antibody. Verified clones were then stored in liquid nitrogen in DMEM medium supplemented as described above, with the addition of an additional 10% FBS and 10% DMSO, for future studies.

### 4.12 PHGDH stability in U251 cell line

U251 cells stably expressing wild‐type, K289Q, or K289R PHGDH proteins were seeded at 2.5 × 10^5^ in 6‐well plates and grown to ~80% confluence. Cells were then treated with cycloheximide (Sigma, C4859) at a final concentration of 75 μg/mL to inhibit de novo protein synthesis, and samples were collected at different time points (0, 3, 6, 9, 24, and 30 h) following treatment. Control treatment was done by adding DMSO. At each time point, cells were washed with phosphate‐buffered saline (PBS), detached with EDTA trypsin, and the cellular pellet resuspended in sample buffer at a final concentration of 0.5 × 10^4^ cells/μL. Samples were heated at 99°C for 10 min. Equal amounts of cells (2 × 10^5^ cells) were loaded onto a SDS‐PAGE gel and proteins were then transferred onto PVDF membranes for Western blot analyses. Membranes were probed with antibodies against flag‐PHGDH and GAPDH (as a loading control), followed by fluorescence‐conjugated secondary antibodies and detection by enhanced fluorescence (Table S7). Band intensities were quantified using Image Studio 6.0, normalized to GAPDH, and expressed as a fraction of the t = 0 value. The relative protein abundance at each time point was plotted against time.

### 4.13 Confocal analyses

To assess the subcellular localization of the PHGDH proteins, p‐formaldehyde‐fixed transfected U251‐PHGDH wild‐ type and variants, as well as U251 non‐transfected cells, were fixed in 4% p‐formaldehyde in phosphate‐buffer saline (PBS), permeabilized, and the unspecific binding sites were blocked by incubation in PBS supplemented with 0.2% Triton X‐100 and 4% horse serum. Subsequently, to evaluate the distributions of PHGDH variants relatively to the endogenous PHGDH wild‐type enzyme, double immunostainings were performed using a mixture of primary antibodies consisting of the polyclonal rabbit anti‐FLAG antibody along with the monoclonal mouse anti‐PHGDH (Table S7). Cells were incubated with primary antibodies overnight at 4°C and, after extensive washing in PBS supplemented with 1% horse serum, anti‐rabbit Alexa 546 antibody (1:1000, ThermoScientific, Waltham, MA) and anti‐mouse Alexa 488 antibody (1:1000, ThermoScientific, Waltham, MA) diluted in PBS, 1% Triton X‐100. Finally, the slides were mounted using a medium containing DAPI stain for preservation. Negative controls were performed by omitting primary antibodies and using U251 untransfected control cells. Immunostained coverslips were imaged using an inverted laser scanning confocal Nikon microscope (Nikon A1 R Laser Scanning confocal), equipped with a ×60 oil immersion objective (NA 1.4). Confocal images were acquired using a 405 nm laser line and a 450/50 Emission filter for DAPI visualization, a 488 nm laser line and a 525/50 emission filter for Alexa 488 visualization, and a 561 nm laser line and a 595/50 emission filter for Alexa 546 visualization. Moreover, acquisition was performed in a sequential modality to avoid interference between each channel due to spectral overlap and without saturating any pixel. Based on previous evidence (Ma et al., 2021), confocal analyses were first exploited to evaluate a partial nuclear localization of PHGDH variants. Particularly, for each transfected cell, the surface delimited by the signal corresponding to the ectopically expressed PHGDH variants and that corresponding to the nucleus were defined as regions of interest (ROI). The respective areas (expressed as ${\mathrm{\mu m}}^{2}$) and Mean Gray Values were measured in the red channel, where the emission of fluorophore associated with the recombinant protein was monitored. Subsequently, a 3D analysis of signal distribution was performed by approximating both cells and nuclei to spheres, see (Sacchi et al., 2025). Second, to further examine changes in the subcellular localization of the PHGDH variants, images from co‐immunostained slides were processed to compare the distribution of the recombinant protein with that of the endogenous (wild‐type) form, considering that the U251 cell line endogenously expresses the PP's enzymes.

## AUTHOR CONTRIBUTIONS

**Elena Zerbini:** Conceptualization; investigation; writing – original draft; methodology; validation; visualization; writing – review and editing; formal analysis; data curation. **Daniele Riva:** Investigation; writing – original draft; methodology; validation; visualization; writing – review and editing; formal analysis; data curation. **Elisa Maffioli:** Investigation; writing – original draft; methodology; validation; writing – review and editing; data curation; formal analysis. **Gabriella Tedeschi:** Conceptualization; investigation; funding acquisition; writing – original draft; methodology; validation; writing – review and editing; formal analysis; data curation. **Silvia Sacchi:** Conceptualization; investigation; writing – original draft; methodology; validation; visualization; writing – review and editing; formal analysis; supervision; data curation. **Loredano Pollegioni:** Conceptualization; funding acquisition; writing – original draft; methodology; validation; writing – review and editing; formal analysis; project administration; supervision.

## CONFLICT OF INTEREST STATEMENT

The authors declare no conflict of interest.

## Supporting information


**Table S1.** List of hippocampal samples.
**Table S2.** Prediction of post‐translational modifications of PHGDH.
**Table S3.** Conditions used for the expression of PHGDH wild‐type and variants in *E. coli* cells.
**Table S4.** Secondary structure of human PHGDH.
**Table S5.** Estimation of subcellular distribution of PHGDH variants based on immunofluorescence signals.
**Table S6.** Estimation of subcellular distribution of PHGDH variants based on normalized fluorescence signals.
**Table S7.** Primary and secondary antibodies, with relative dilutions and incubation protocols.
**Table S8.** Forward and reverse primers for the expression of PHGDH variants in *Escherichia coli*.
**Table S9.** Forward and reverse primers for the over‐expression of PHGDH variants in mammalian cells.
**Figure S1.** Primary sequence of human PHGDH.
**Figure S2.** Representative MS^2^ spectra and extracted‐ion chromatograms (XICs) of the transitions observed for PHGDH phosphopeptides.
**Figure S3.** SDS‐PAGE analysis of all purified PHGDH variants.
**Figure S4.** Thermostability of PHGDH variants.
**Figure S5.** Comparison of CD spectra of the PHGDH variants.
**Figure S6.** Effect of NADH binding.
**Figure S7.** Effect of NAD^+^ binding.
**Figure S8.** Prediction of subcellular localization of wild‐type and variant PHGDH proteins.
**Figure S9.** Cycloheximide chase analysis of protein stability in U251 cells.
**Figure S10.** Tetrameric arrangement of the predicted PHGDH structure.
**Figure S11.** Correlation analyses between PHGDH quaternary assembly and biochemical parameters.
Scatter plots show the relationship between SEC‐derived tetramer abundance and biochemical and structural readouts for PHGDH variants (**wild‐type**, **S55A**, **S55D**, **T60A**, **T60D**, **T78A**, **T78D**, **S383A, S383D, S473A, S473D, K289Q**, **K289R**; *n* = 13). Tetramer abundance was quantified at four protein concentrations (0.33, 1, 3.33, and 10 mg/mL) and compared with catalytic turnover (k_cat_, panel A), catalytic efficiency (k_cat_/K_M_, panel B), thermal stability (T_m_, panel C), α‐helical content (panel D), and total structured secondary‐structure content (α‐helix + β‐sheet, panel E). Spearman rank correlation coefficients (ρ) are reported for each comparison. No monotonic associations were detected for T_m_ (ρ = −0.005 to −0.110), α‐helical content (ρ = −0.053 to 0.112), and α‐helix + β‐sheet content (ρ = −0.104 to 0.088) across the tested protein concentration range. Catalytic efficiency showed no monotonic association with tetramer abundance at 1–10 mg/mL (ρ ≈ 0), whereas a moderate negative correlation was observed at 0.33 mg/mL (ρ = −0.407). Catalytic turnover (k_cat_) exhibited a stronger negative correlation with baseline tetramer abundance at 0.33 mg/mL (ρ = −0.68), which progressively weakened at higher protein concentrations and was absent at 10 mg/mL. To assess concentration‐dependent oligomerization, the slope of % tetramer versus log_10_(protein concentration) (1–10 mg/mL) was calculated and compared with the same parameters (panel F), yielding only weak monotonic associations (ρ = −0.174 to 0.193). No monotonic association was observed between concentration‐dependent tetramer loss (slope) and catalytic turnover (ρ = 0.754). All statistical analyses were performed using Spearman's rank correlation coefficient.

## Data Availability

The data that support the findings of this study are openly available in ProteomeXchange Consortium at https://www.proteomexchange.org/, reference number PXD072965.
