# Supplementary material for: Post‐translational regulation of human D‐3‐phosphoglycerate dehydrogenase in Alzheimer's disease
Source: Protein Sci. 2026 Feb 19;35(3):e70505. doi: 10.1002/pro.70505 (PMC12917857; doi:10.1002/pro.70505)
Supplement: Supplementary file 1 — Table S1. List of hippocampal samples. Table S2. Prediction of post‐translational modifications of PHGDH. Table S3. Conditions used for the expression of PHGDH wild‐type and variants in E. coli cells. Table S4. Secondary structure of human PHGDH. Table S5. Estimation of subcellular distribution of PHGDH variants based on immunofluorescence signals. Table S6. Estimation of subcellular distribution of PHGDH variants based on normalized fluorescence signals. Table S7. Primary and secondary antibodies, with relative dilutions and incubation protocols. Table S8. Forward and reverse primers for the expression of PHGDH variants in Escherichia coli. Table S9. Forward and reverse primers for the over‐expression of PHGDH variants in mammalian cells. Figure S1. Primary sequence of human PHGDH. Figure S2. Representative MS2 spectra and extracted‐ion chromatograms (XICs) of the transitions observed for PHGDH phosphopeptides. Figure S3. SDS‐PAGE analysis of all purified PHGDH variants. Figure S4. Thermostability of PHGDH variants. Figure S5. Comparison of CD spectra of the PHGDH variants. Figure S6. Effect of NADH binding. Figure S7. Effect of NAD+ binding. Figure S8. Prediction of subcellular localization of wild‐type and variant PHGDH proteins. Figure S9. Cycloheximide chase analysis of protein stability in U251 cells. Figure S10. Tetrameric arrangement of the predicted PHGDH structure. Figure S11. Correlation analyses between PHGDH quaternary assembly and biochemical parameters. Scatter plots show the relationship between SEC‐derived tetramer abundance and biochemical and structural readouts for PHGDH variants (wild‐type, S55A, S55D, T60A, T60D, T78A, T78D, S383A, S383D, S473A, S473D, K289Q, K289R; n = 13). Tetramer abundance was quantified at four protein concentrations (0.33, 1, 3.33, and 10 mg/mL) and compared with catalytic turnover (kcat, panel A), catalytic efficiency (kcat/KM, panel B), thermal stability (Tm, panel C), α‐helical content (panel D), and total structured s [file PRO-35-e70505-s001.docx]

# SUPPLEMENTARY MATERIALS

**Table S1. List of hippocampal samples.**

List of CTR and AD samples used for the investigation of PHGHD’s PTMs, along with the corresponding enzyme expression levels, indicated as ng of PHGDH per µg of total proteins. Values are reported as mean ± SEM (n = 3).

|  | **CTR** | | | | **AD** | | | |
| --- | --- | --- | --- | --- | --- | --- | --- | --- |
|  | **Sample** | **Expired age** | **PMI** | **ng/μg total proteins** | **Sample** | **Expired age** | **PMI** | **ng/μg total proteins** |
| **Male** | A297/16 A134/00 A264/18 A114/12  A053/11  A297/13 | 81  86  90  82  77  - | 10  6  12  24  11  - | 0.540 ± 0.035 | A186/04 A283/09 A267/13 A282/11  A277/15 | 71  77  76  93  87 | 5.3  9.7  11  13.5  6 | 0.701 ± 0.031 |
| **Female** | A051/14 A083/18 A359/08 A144/10 A158/14 A046/18  A407/13  A402/17 | 76  81  80  92  73  81  80  78 | 22  26.5  3  22.5  27  27  22  6 | 0.519 ± 0.031 | A204/11 A219/07 A032/13 A163/15 A169/10 A219/07  A272/15  A092/15 | 92  82  86  76  85  82  90  86 | 11  4.5  12  4  10  4.5  10  13 | 0.923 ± 0.125 |

**Table S2. Prediction of post-translational modifications of PHGDH.**

Prediction of **a)** phosphorylation and **b)** acetylation sites within PHGDH using multiple online bioinformatic tools: NetPhos 3.1 (<https://services.healthtech.dtu.dk/services/NetPhos-3.1/>); PhosphoSitePlus (<https://www.phosphosite.org/homeAction.action>);  PhosphoNET ([http://www.phosphonet.ca](http://www.phosphonet.ca/)); GPS ([http://gps.biocuckoo.cn](http://gps.biocuckoo.cn/)); PTMfunc (<http://ptmfunc.com/>). Residues experimentally identified as modified in this work are highlighted in green.

|  |  | **K8** | **S12** | **S14** | **K21** | **K33** | **S37** | **K38** | **S55** | **T57** | **K58** | **T60** | **K69** | **T78** | **T89** | **K91** | **T98** | **S103** | **S105** | **T110** | **T125** | **S127** | **K129** | **K132** | **K136** | **K137** | **T141** | **K146** | **T147** | **T162** | **S166** | **K170** | **T171** | **Y174** | **S179** | **S183** | **S185** | **T204** |
| --- | --- | --- | --- | --- | --- | --- | --- | --- | --- | --- | --- | --- | --- | --- | --- | --- | --- | --- | --- | --- | --- | --- | --- | --- | --- | --- | --- | --- | --- | --- | --- | --- | --- | --- | --- | --- | --- | --- |
| **a)** | NetPhos 3.1 |  | × | × |  |  | × |  | × | × |  | × |  | × |  |  |  | × | × |  |  | × |  |  |  |  |  |  | × |  | × |  |  |  | × | × |  |  |
|  | PhosphoSite |  |  | × |  |  |  |  | × | × |  | × |  | × |  |  |  |  | × |  | × | × |  |  |  |  | × |  | × |  |  |  |  |  |  |  |  |  |
|  | PhosphoNET |  |  | × |  |  | × |  |  |  |  |  |  | × | × |  | × |  | × |  | × | × |  |  |  |  | × |  |  |  | × |  |  | × | × |  |  |  |
|  | GPS |  |  |  |  |  | × |  |  |  |  |  |  | × |  |  |  |  |  | × | × |  |  |  |  |  |  |  |  |  |  |  | × |  |  |  |  |  |
|  | PTMfunc |  |  |  |  |  |  |  | × | × |  | × |  |  |  |  |  |  |  |  | × | × |  |  |  |  |  |  |  |  |  |  |  |  |  |  |  |  |
| **b)** | PhosphoSite |  |  |  |  | × |  |  |  |  | × |  |  |  |  |  |  |  |  |  |  |  |  |  |  |  |  |  |  |  |  |  |  |  |  |  |  |  |
|  | PTMfunc |  |  |  | × |  |  |  |  |  |  |  |  |  |  |  |  |  |  |  |  |  |  |  |  |  |  |  |  |  |  |  |  |  |  |  |  |  |

|  |  | **T207** | **S212** | **T213** | **T214** | **T221** | **K226** | **K227** | **S251** | **T263** | **S280** | **S287** | **T288** | **K289** | **S293** | **K308** | **K310** | **S311** | **T313** | **T322** | **S323** | **S326** | **T329** | **K330** | **T341** | **S349** | **K351** | **T353** | **T358** | **T361** | **S362** | **K364** | **S371** | **K380** | **S383** | **K384** | **K394** | **K398** |
| --- | --- | --- | --- | --- | --- | --- | --- | --- | --- | --- | --- | --- | --- | --- | --- | --- | --- | --- | --- | --- | --- | --- | --- | --- | --- | --- | --- | --- | --- | --- | --- | --- | --- | --- | --- | --- | --- | --- |
| **a)** | NetPhos 3.1 |  |  |  |  |  |  |  | × |  | × | × |  |  | × |  |  |  |  | × | × | × | × |  | × | × |  |  |  | × | × |  | × |  |  |  |  |  |
|  | PhosphoSite |  |  |  |  |  |  |  |  |  |  |  |  |  |  |  |  |  |  |  |  |  |  |  |  | × |  | × | × |  | × |  | × |  |  |  |  |  |
|  | PhosphoNET |  |  |  |  | × |  |  |  | × | × | × |  |  | × |  |  |  |  |  |  | × |  |  |  | × |  | × | × |  |  |  | × |  | × |  |  |  |
|  | GPS |  |  | × | × |  |  |  | × |  |  |  | × |  |  |  |  |  |  |  |  |  |  |  |  |  |  |  |  |  |  |  |  |  |  |  |  |  |
|  | PTMfunc |  |  |  |  |  |  |  |  |  |  |  |  |  |  |  |  |  |  |  |  |  |  |  |  |  |  |  |  |  |  |  | × |  |  |  |  |  |
| **b)** | PhosphoSite |  |  |  |  |  |  |  |  |  |  |  |  | × |  |  |  |  |  |  |  |  |  |  |  |  | × |  |  |  |  |  |  |  |  |  | × |  |
|  | PTMfunc |  |  |  |  |  |  |  |  |  |  |  |  |  |  |  |  |  |  |  |  |  |  |  |  |  |  |  |  |  |  |  |  |  |  |  |  |  |

|  |  | **T405** | **T406** | **S407** | **S409** | **Y432** | **T441** | **T442** | **T470** | **T472** | **S473** | **T480** | **S494** | **Y495** | **T497** | **S498** | **S501** | **T505** | **S512** | **S513** | **S517** | **K522** | **T526** |
| --- | --- | --- | --- | --- | --- | --- | --- | --- | --- | --- | --- | --- | --- | --- | --- | --- | --- | --- | --- | --- | --- | --- | --- |
| **a)** | NetPhos 3.1 |  |  | × | × |  |  | × |  | × | × |  | × |  |  |  | × | × |  |  | × |  |  |
|  | PhosphoSite |  |  |  |  |  |  |  |  |  | × | × |  |  |  |  |  |  |  |  |  |  |  |
|  | PhosphoNET | × |  | × | × |  |  |  |  |  |  | × |  |  |  | × | × |  |  |  | × |  |  |
|  | GPS |  |  |  |  |  |  |  |  |  |  |  |  |  | × |  |  |  |  |  |  |  |  |
|  | PTMfunc |  |  |  |  |  |  |  |  |  |  | × |  |  |  |  |  |  |  |  |  |  |  |
| **b)** | PhosphoSite |  |  |  |  |  |  |  |  |  |  |  |  |  |  |  |  |  |  |  |  |  |  |
|  | PTMfunc |  |  |  |  |  |  |  |  |  |  |  |  |  |  |  |  |  |  |  |  |  |  |

**Table S3. Conditions used for the expression of PHGDH wild-type and variants in *E. coli* cells.**

All proteins were expressed in soluble form. Their relative expression levels and purification yields are reported below. LB, Luria Bertani broth; TB, Terrific broth.

| **PHGDH** **variants** | **Cultivation** **broth** | **OD_600 nm_ at induction** | **IPTG (mM)** | **Temperature after induction ( °C)** | **Time of collection (h)** | **Expression level (mg/L)** | **Purification level (mg/g_cells_)** |
| --- | --- | --- | --- | --- | --- | --- | --- |
| **Wild-type** | LB | 0.6 | 0.5 | 20 | 20 | 95 | 9.0 |
| **S55A** | TB |  | 0.01 | 17 |  | 24.9 | 2.6 |
| **S55D** |  |  |  |  |  | 14.2 | 1.2 |
| **T60A** |  |  |  |  |  | 16.3 | 1.9 |
| **T60D** |  |  |  |  |  | 21.6 | 2.5 |
| **T78A** |  |  |  |  |  | 23.5 | 1.6 |
| **T78D** |  |  |  |  |  | 7.8 | 1.7 |
| **S383A** |  |  |  |  |  | 3.5 | 1.2 |
| **S383D** |  |  |  |  |  | 8.5 | 1.4 |
| **S473A** |  |  |  |  |  | 3.0 | 1.1 |
| **S473D** |  |  |  |  |  | 6.5 | 1.3 |
| **K289Q** |  |  |  |  |  | 4.2 | 1.2 |
| **K289R** |  |  |  |  |  | 4.7 | 1.7 |

**Table S4. Secondary structure of human PHGDH.**

Melting temperatures and secondary structure elements content (estimated by deconvoluting far-UV CD spectra through DichroWeb software) of wild-type and variants of PHGDH. Values are reported as mean ± SEM (n = 3).

| **Variants** | **T_m_ ( °C)** | **α-helix (%)** | **β-sheets (%)** | | |
| --- | --- | --- | --- | --- | --- |
| **Wild-type** | 45.2 ± 0.9 | 33 | | 16 |  |
| **S55A** | 41.2 ± 1.1 | 30 | | 17 |  |
| **S55D** | 48.2 ± 1.3 | 34 | | 16 |  |
| **T60A** | 40.5 ± 1.2 | 28 | | 19 |  |
| **T60D** | 43.3 ± 1.4 | 29 | | 16 |  |
| **T78A** | 46.1 ± 0.4 | 31 | | 16 |  |
| **T78D** | 45.6 ± 1.9 | 33 | | 16 |  |
| **S383A** | 43.7 ± 0.4 | 31 | | 17 |  |
| **S383D** | 44.9 ± 0.4 | 32 | | 16 |  |
| **S473A** | 41.9 ± 0.1 | 27 | | 20 |  |
| **S473D** | 43.5 ± 1.3 | 31 | | 17 |  |
| **K289Q** | 42.7 ± 0.9 | 28 | | 17 |  |
| **K289R** | 46.9 ± 2.9 | 33 | | 17 |  |

**Table S5. Estimation of subcellular distribution of PHGDH variants based on immunofluorescence signals.**

For each PHGDH variant, the nuclear and cytosolic fractions were statistically compared with those of the wild-type enzyme using Dunn’s post-hoc test following a Kruskal–Wallis test, the non-parametric analog of a one-way ANOVA.

| **PHGDH** | **Analyzed cells (number)** | **Nuclear fraction (%)** | **st. dev.** | **Cytosolic fraction (%)** | **st. dev.** | **Dunn’s test (*vs*wt)**  **Adjusted P value** |
| --- | --- | --- | --- | --- | --- | --- |
| **wt** | 59 | 6.9 | 3.6 | 93.1 | 3.6 |  |
| **S55A** | 31 | 7.8 | 5.2 | 92.2 | 5.2 | >0.9999 |
| **S55D** | 30 | 9.2 | 5.4 | 90.8 | 5.4 | >0.9999 |
| **T60A** | 26 | 11.1 | 6.6 | 88.9 | 6.6 | 0.0379 |
| **T60D** | 21 | 9.7 | 5.2 | 90.3 | 5.2 | 0.5261 |
| **T78A** | 36 | 8.8 | 6.1 | 91.2 | 6.1 | >0.9999 |
| **T78D** | 38 | 9.3 | 6.2 | 90.7 | 6.2 | >0.9999 |
| **S371A** | 43 | 6.4 | 4.5 | 93.6 | 4.5 | >0.9999 |
| **S371D** | 61 | 7.0 | 4.4 | 93.0 | 4.4 | >0.9999 |
| **S383A** | 73 | 9.3 | 6.1 | 90.7 | 6.1 | >0.9999 |
| **S383D** | 49 | 8.9 | 6.9 | 91.1 | 6.9 | >0.9999 |
| **S473A** | 43 | 7.2 | 5.2 | 92.7 | 5.2 | >0.9999 |
| **S473D** | 40 | 7.9 | 5.7 | 92.1 | 5.7 | >0.9999 |
| **K289Q** | 35 | 5.9 | 2.5 | 94.1 | 2.5 | >0.9999 |
| **K289R** | 43 | 6.0 | 4.6 | 94.0 | 4.6 | 0.2596 |

**Table S6. Estimation of subcellular distribution of PHGDH variants based on normalized fluorescence signals.**

Before estimating the subcellular distribution, signals corresponding to the overall protein content and its nuclear fraction were normalized to the respective mean values. More in detail, normalization was performed independently between whole cells and the nuclei, using the mean expression levels indicated by the Mean Gray Value (mgv) parameter. Subsequently, as before, for each PHGDH variant, both the nuclear and cytosolic fractions were statistically compared to those obtained for the wild-type enzyme using the post-hoc Dunn’s test following a Kruskal–Wallis test, the non-parametric analog of a one-way ANOVA.

| **PHGDH** | **Analyzed cells (number)** | **Total ectopic expression (mgv)** | **Nuclear fraction**  **(%)** | **st. dev.** | **Cytosolic fraction**  **(%)** | **st. dev.** | **Dunn’s test (*vs* wt)**  **Adjusted P value** |
| --- | --- | --- | --- | --- | --- | --- | --- |
| **wt** | 59 | 784.2 | 8.7 | 3.5 | 91.3 | 3.5 |  |
| **S55A** | 31 | 1491.3 | 7.4 | 3.9 | 92.6 | 3.9 | 0.9855 |
| **S55D** | 30 | 1189.6 | 8.7 | 4.2 | 91.3 | 4.2 | >0.9999 |
| **T60A** | 26 | 1644.4 | 9.2 | 4.0 | 90.8 | 4.0 | >0.9999 |
| **T60D** | 21 | 858.7 | 8.3 | 4.5 | 91.7 | 4.5 | >0.9999 |
| **T78A** | 36 | 1126.0 | 8.1 | 4.6 | 91.9 | 4.6 | >0.9999 |
| **T78D** | 38 | 1162.9 | 8.0 | 3.6 | 92.0 | 3.6 | >0.9999 |
| **S371A** | 43 | 690.7 | 7.1 | 3.5 | 92.9 | 3.5 | 0.1923 |
| **S371D** | 61 | 697.8 | 7.1 | 3.4 | 92.9 | 3.4 | 0.0593 |
| **S383A** | 73 | 1175.2 | 8.5 | 4.2 | 91.5 | 4.2 | >0.9999 |
| **S383D** | 49 | 1165.6 | 8.5 | 5.3 | 91.5 | 5.3 | >0.9999 |
| **S473A** | 43 | 969.0 | 7.5 | 3.2 | 92.5 | 3.2 | >0.9999 |
| **S473D** | 40 | 894.4 | 8.7 | 5.5 | 91.3 | 5.5 | >0.9999 |
| **K289Q** | 35 | 360.7 | 7.4 | 4.0 | 92.6 | 4.0 | 0.5929 |
| **K289R** | 43 | 340.5 | 6.4 | 3.3 | 93.6 | 3.3 | 0.0008 |

# Table S7. Primary and secondary antibodies, with relative dilutions and incubation protocols.

# Antibodies used for the detection of PHGDH in a) hippocampal tissue lysates to evaluate its endogenous expression levels, b) IP experiments to verify its presence in eluted fractions, c) protein stability studies to evaluate half-life of PHGDH variants through Western blot analysis, and d) immunofluorescence studies to determine the localization of the wild-type protein and variants. RT, room temperature; O.N., overnight.

| **Exp.** | **Primary antibodies** | **Dilution** | **Protocol** |
| --- | --- | --- | --- |
| **a)** | Rabbit anti-PHGDH antibody (HPA024031, Sigma) | 1:1000 | 2 h, RT |
|  | Mouse anti-GAPDH antibody (MA5-15738, Invitrogen) | 1:2000 | 2 h, RT |
| **b)** | Mouse anti-PHGDH antibody (sc-100317, Santa Cruz) | 1:1000 | O.N., 4 °C |
| **c)** | Rabbit anti-FLAG antibody (F7425, Sigma) | 1:500 | 2 h, RT |
|  | Mouse anti-GAPDH antibody (MA5-15738, Invitrogen) | 1:2000 | 2 h, RT |
| **d)** | Rabbit anti-FLAG antibody (F7425, Sigma) | 1:500 | O.N., 4 °C |
|  | Mouse anti-PHGDH antibody (mouse anti-serum dialyzed; DAVIDS Biotechnologie) | 1:250 | O.N., 4 °C |
|  | **Secondary antibodies** |  |  |
| **a)** | Anti-rabbit IgG (H+L) Alexa Fluor Plus800 (A32735, Invitrogen) | 1:20000 | 1 h, RT |
|  | Anti-Mouse IRDye 680RD (926-68070, LI-COR Biosciences) | 1:5000 | 1 h, RT |
| **b)** | Donkey anti-mouse IgG antibody (715-035-150, Jackson ImmunoResearch) | 1:10000 | 1 h, RT |
| **c)** | Anti-rabbit IgG (H+L) Alexa Fluor Plus800 (A32735, Invitrogen) | 1:20000 | 1 h, RT |
|  | Anti-Mouse IRDye 680RD (926-68070, LI-COR Biosciences) | 1:5000 | 1 h, RT |
|  | Anti-rabbit Alexa 546 antibody (A-31573, ThermoScientific) | 1:1000 | 1 h, RT |
|  | Anti-mouse Alexa 488 antibody (A-21202, ThermoScientific) | 1:1000 | 1 h, RT |

# Table S8. Forward and reverse primers for the expression of PHGDH variants in *Escherichia coli*.

# Primers designed for the site-specific mutagenesis reactions within pETM11-His-hPHGDH construct: in bold and highlighted in yellow the mismatched nucleotides.

| **Variant** | **Strand** | **Sequence** |
| --- | --- | --- |
| **S55A** | **Forward** | 5’—GTCTGATCGTTCGC**GC**CGCGACCAAAGTGACCG—3’ |
|  | **Reverse** | 5’—CGGTCACTTTGGTCGCG**GC**GCGAACGATCAGAC—3’ |
| **S55D** | **Forward** | 5’—GATCGTTCGC**GA**CGCGACCAAAGTGACCGC—3’ |
|  | **Reverse** | 5’—GCGGTCACTTTGGTCGCG**TC**GCGAACGATC—3’ |
| **T60A** | **Forward** | 5’—GCGACCAAAGTG**G**CCGCTGATGTGATTAACG—3’ |
|  | **Reverse** | 5’—CGTTAATCACATCAGCGG**C**CACTTTGGTCGC—3’ |
| **T60D** | **Forward** | 5’—GCGCGACCAAAGTG**GA**CGCTGATGTGATTAACGCAGC—3’ |
|  | **Reverse** | 5’—GCTGCGTTAATCACATCAGCG**TC**CACTTTGGTCGCGC—3’ |
| **T78A** | **Forward** | 5’—GTAGTTGGTCGTGCAGGC**G**CAGGTGTTGATAATGTTGACC—3’ |
|  | **Reverse** | 5’—GGTCAACATTATCAACACCTG**C**GCCTGCACGACCAACTAC—3’ |
| **T78D** | **Forward** | 5’—GTTGGTCGTGCAGGC**GAC**GGTGTTGATAATGTTGACCTGGAAG—3’ |
|  | **Reverse** | 5’—CTTCCAGGTCAACATTATCAACACC**GTC**GCCTGCACGACCAAC—3’ |
| **S383A** | **Forward** | 5’—GCCTGCTGAAAGAAGCA**G**CTAAACAGGCCGATG—3’ |
|  | **Reverse** | 5’—CATCGGCCTGTTTAG**C**TGCTTCTTTCAGCAGGC—3’ |
| **S383D** | **Forward** | 5’—GGCCTGCTGAAAGAAGCA**GA**TAAACAGGCCGATGTTAATCTG—3’ |
|  | **Reverse** | 5’—CAGATTAACATCGGCCTGTTTA**TC**TGCTTCTTTCAG-CAGGCC—3’ |
| **S473A** | **Forward** | 5’—CGTACCCAAACC**G**CAGATCCGGCAATGCTGC—3’ |
|  | **Reverse** | 5’—GCAGCATTGCCGGATCTG**C**GGTTTGGGTACG—3’ |
| **S473D** | **Forward** | 5’—GTTTCGTACCCAAACC**GAC**GATCCGGCAATGCTGCCTACC—3’ |
|  | **Reverse** | 5’—GGTAGGCAGCATTGCCGGATC**GTC**GGTTTGGGTACGAAAC—3’ |
| **K289A** | **Forward** | 5’—CATCTGGGTGCAAGCACC**GC**AGAAGCTCAAAGCCGTTG—3’ |
|  | **Reverse** | 5’—CAACGGCTTTGAGCTTCT**GC**GGTGCTTGCACCCAGATG—3’ |
| **K289Q** | **Forward** | 5’—ATCTGGGTGCAAGCACC**C**A**G**GAAGCTCAAAGCCGTTG—3’ |
|  | **Reverse** | 5’—CAACGGCTTTGAGCTTC**C**T**G**GGTGCTTGCACCCAGAT—3’ |
| **K289R** | **Forward** | 5’—TCTGGGTGCAAGCACCA**G**AGAAGCTCAAAGCC—3’ |
|  | **Reverse** | 5’—GGCTTTGAGCTTCT**C**TGGTGCTTGCACCCAGA—3’ |

# Table S9. Forward and reverse primers for the over-expression of PHGDH variants in mammalian cells.

Primers designed for the site-specific mutagenesis reactions within pcDNA3.1+/C-(K)-DYK-hPHGDH-1xFLAG construct: in bold and highlighted in yellow the mismatched nucleotides.

| **Variant** | **Strand** | **Sequence** |
| --- | --- | --- |
| **S55A** | **Forward** | 5’—GGCCTTATTGTTCGC**G**CTGCCACCAAGGTGA—3’ |
|  | **Reverse** | 5’—TCACCTTGGTGGCAG**C**GCGAACAATAAGGCC—3’ |
| **S55D** | **Forward** | 5’—AAGGCCTTATTGTTCGC**GA**TGCCACCAAGGTGACCG—3’ |
|  | **Reverse** | 5’—CGGTCACCTTGGTGGCA**TC**GCGAACAATAAGGCCTT—3’ |
| **T60A** | **Forward** | 5’—CTGCCACCAAGGTG**G**CCGCTGATGTCATC—3’ |
|  | **Reverse** | 5’—GATGACATCAGCGG**C**CACCTTGGTGGCAG—3’ |
| **T60D** | **Forward** | 5’—GCTCTGCCACCAAGGTG**GA**CGCTGATGTCATCAACG—3’ |
|  | **Reverse** | 5’—CGTTGATGACATCAGCG**TC**CACCTTGGTGGCAGAGC—3’ |
| **T78A** | **Forward** | 5’—GGCAGGGCTGGC**G**CAGGTGTGGACAA—3’ |
|  | **Reverse** | 5’—TTGTCCACACCTG**C**GCCAGCCCTGCC—3’ |
| **T78D** | **Forward** | 5’—GGTGGGCAGGGCTGGC**GAT**GGTGTGGACAATGTGG—3’ |
|  | **Reverse** | 5’—CCACATTGTCCACACC**ATC**GCCAGCCCTGCCCACC—3’ |
| **S383A** | **Forward** | 5’—GGCCTCCTGAAAGAGGCT**G**CCAAGCAGGCG—3’ |
|  | **Reverse** | 5’—CGCCTGCTTGG**C**AGCCTCTTTCAGGAGGCC—3’ |
| **S383D** | **Forward** | 5’—CCTCCTGAAAGAGGCT**GA**CAAGCAGGCGGATGTG—3’ |
|  | **Reverse** | 5’—CACATCCGCCTGCTTG**TC**AGCCTCTTTCAGGAGG—3’ |
| **S473A** | **Forward** | 5’—TCCGGACTCAGACC**G**CTGACCCTGCAATG—3’ |
|  | **Reverse** | 5’—CATTGCAGGGTCAG**C**GGTCTGAGTCCGGA—3’ |
| **S473D** | **Forward** | 5’—TATTCCGGACTCAGACC**GA**TGACCCTGCAATGCTGC—3’ |
|  | **Reverse** | 5’—GCAGCATTGCAGGGTCA**TC**GGTCTGAGTCCGGAATA—3’ |
| **K289A** | **Forward** | 5’—GGGTGCCAGCACC**GC**GGAGGCTCAGAGC—3’ |
|  | **Reverse** | 5’—GCTCTGAGCCTCC**GC**GGTGCTGGCACCC—3’ |
| **K289Q** | **Forward** | 5’—GGGTGCCAGCACC**C**AGGAGGCTCAGAG—3’ |
|  | **Reverse** | 5’—CTCTGAGCCTCCT**G**GGTGCTGGCACCC —3’ |
| **K289R** | **Forward** | 5’—GGGTGCCAGCACCA**G**GGAGGCTCAGAG—3’ |
|  | **Reverse** | 5’—CTCTGAGCCTCC**C**TGGTGCTGGCACCC—3’ |

#
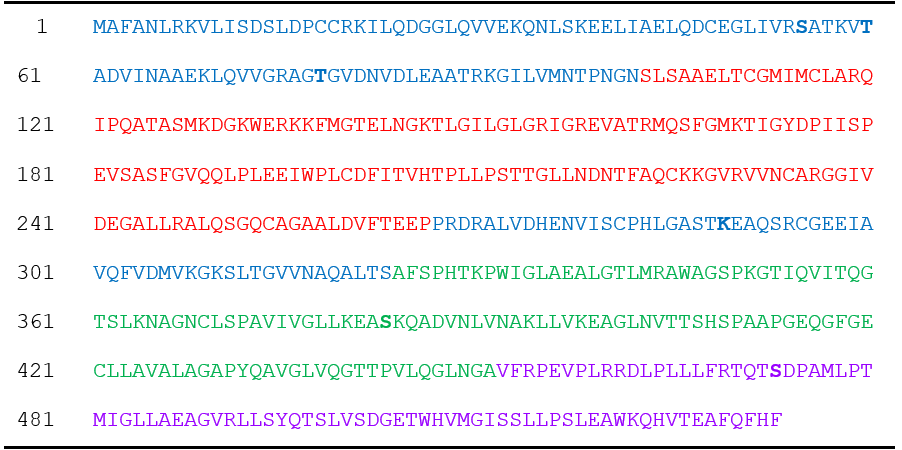


**Figure S1. Primary sequence of human PHGDH.**

Residues experimentally identified as phosphorylated (S55, T60, T78, S383, and S473) or acetylated (K289) in hippocampal samples are shown in bold. Functional domains are color-coded as follows: blue, substrate binding domain (102 aa, 11 kDa - 57 aa, 6 kDa); red, cofactor binding domain (164 aa, 18 kDa); green, ASB (allosteric substrate binding) domain (128 AA, 13 kDa); violet, ACT (Aspartate kinase-Chorismate mutase-TyrA prephenate dehydrogenase) domain (82 AA, 9 kDa).

**
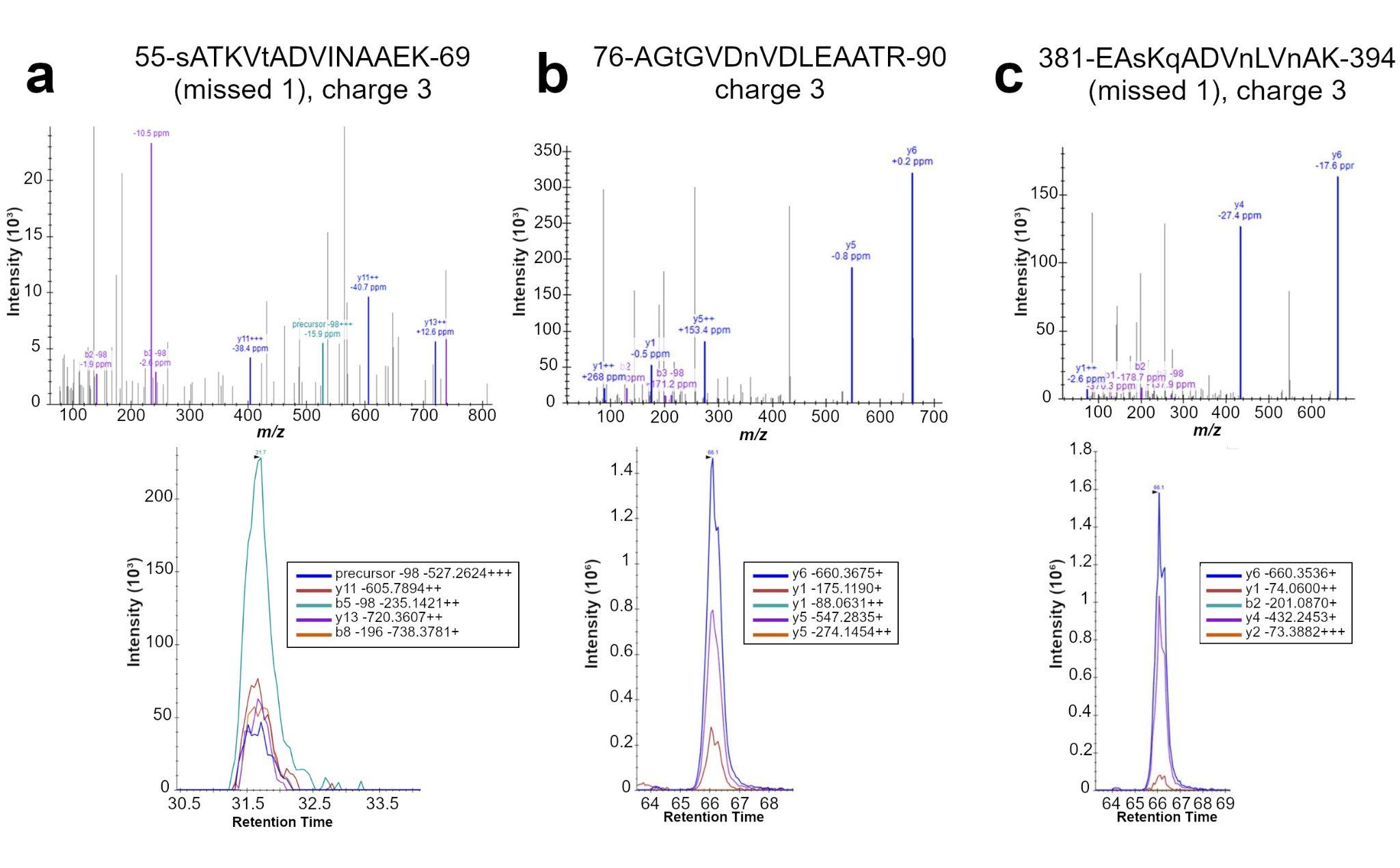
**

**Figure S2. Representative MS² spectra and extracted-ion chromatograms (XICs) of the transitions observed for PHGDH phosphopeptides.**

Panel a) 55-sATKVtADVINAAEK-69; panel b) 76-AGtGVDnVDLEAATR-90; panel c) 381-EAsKqADVnLVnAK-394. Modified sites are indicated in lowercase letters.

**Figure S3. SDS-PAGE analysis of all purified PHGDH variants.**
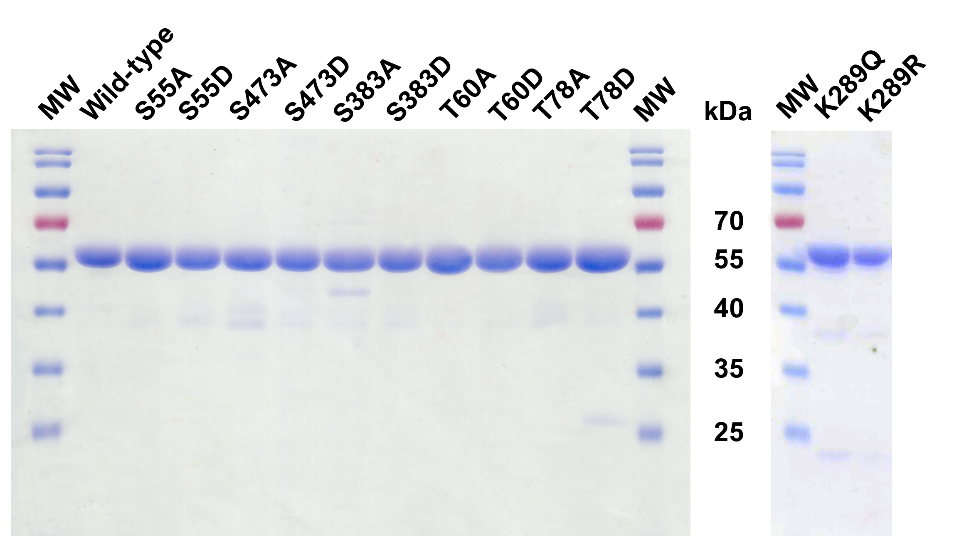


5 µg of each protein variant were loaded onto a 12% acrylamide gel. These included substitutions that mimic phosphorylation (D, Asp) and acetylation (Q, Gln) or prevent phosphorylation (A, Ala) and acetylation (R, Arg).


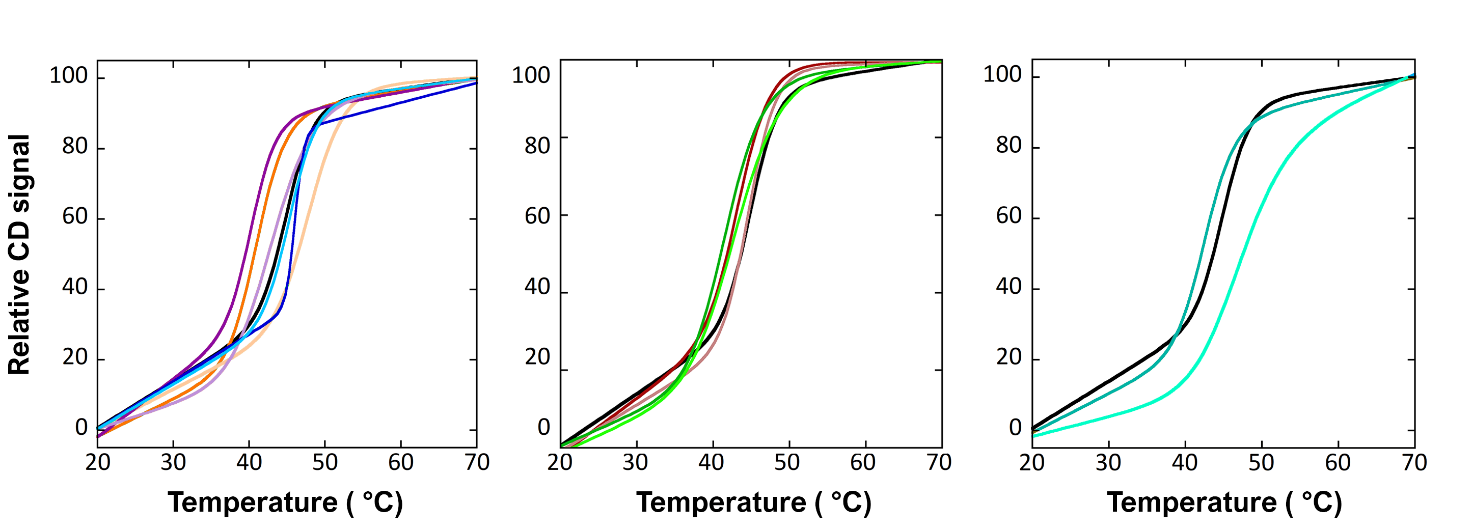


**Figure S4. Thermostability of PHGDH variants.**

Melting temperature profiles of all the phosphorylation/acetylation mimetic PHGDH variants were recorded in a 10 mM phosphate buffer, pH 7.0. Left panel shows thermal unfolding of the variants at the N-terminal end: wild-type (black), S55A (orange), S55D (pale orange), T60A (dark purple), T60D (lavender), T78A (blue), T78D (light blue). The central panel shows the thermal unfolding of the variants at the C-terminal region: wild-type (black), S383A (firebrick), S383D (salmon), S473A (dark green), S473D (light green). The right panel shows the thermal unfolding of the variants at the C-terminal region mimicking the acetylation: wild-type (black), K289Q (dark teal), K289R (aquamarine) (n = 3).

**
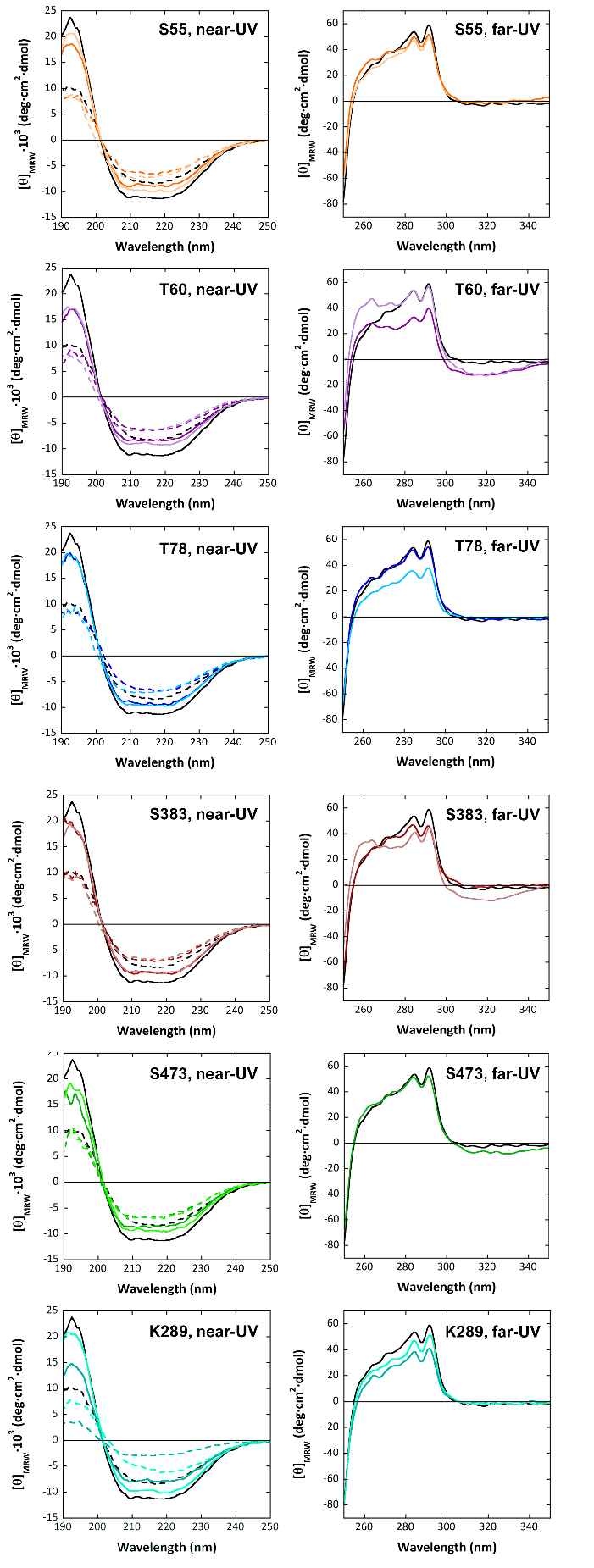
**

**Figure S5. Comparison of CD spectra of the PHGDH variants.**

Near-UV (left panels) and far-UV (right panels) spectra (**—** continuous line, pre-thermal ramp spectrum, **- - -** dashed line, post-thermal ramp spectrum) of PHGDH wild-type and variants: wild-type (black), S55A (orange), S55D (pale orange), T60A (dark purple), T60D (lavender), T78A (blue), T78D (light blue), S383A (firebrick), S383D (salmon), S473A (dark green), S473D (light green), K289Q (dark teal), K289R (aquamarine).


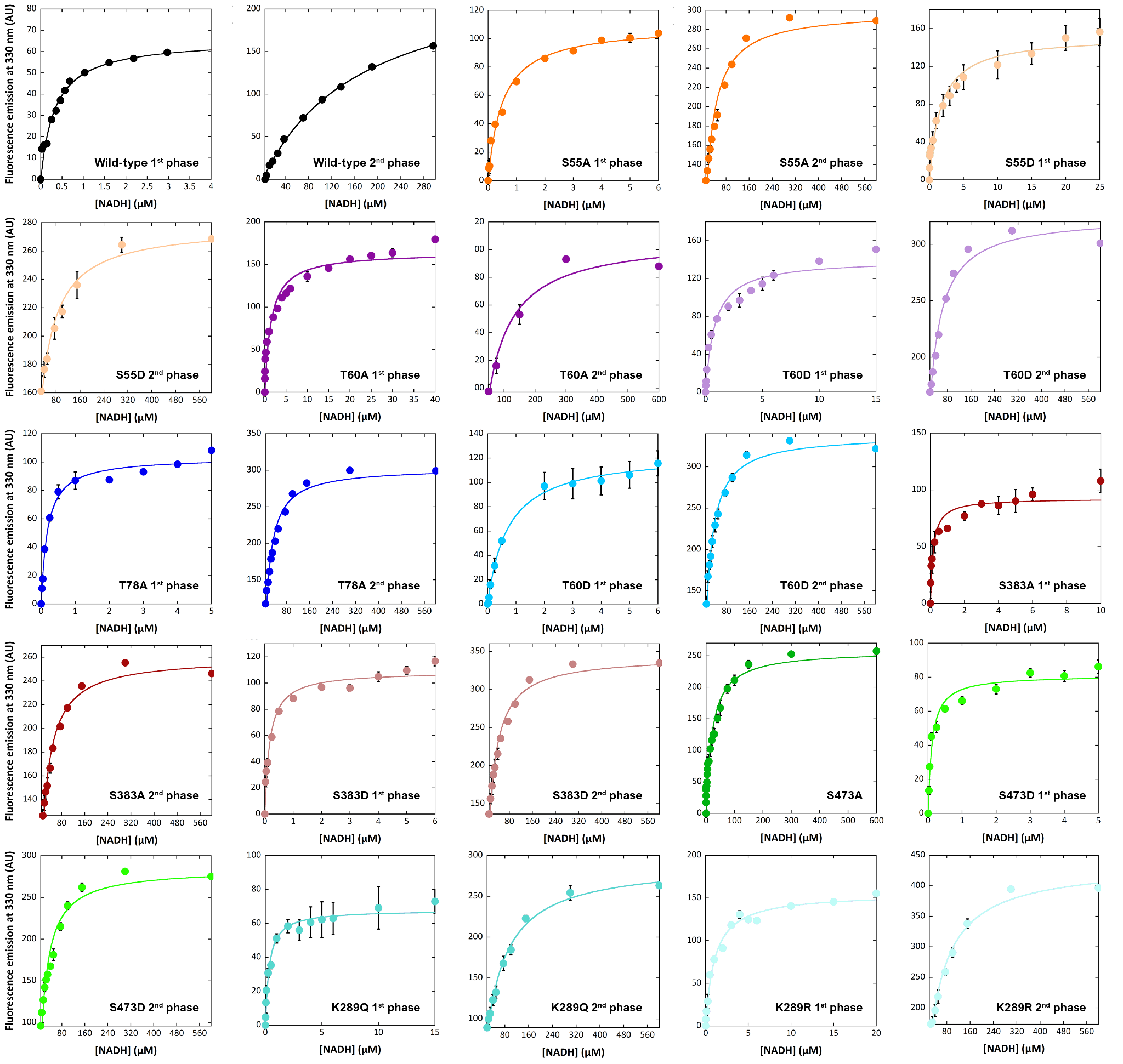


**Figure S6. Effect of NADH binding.**
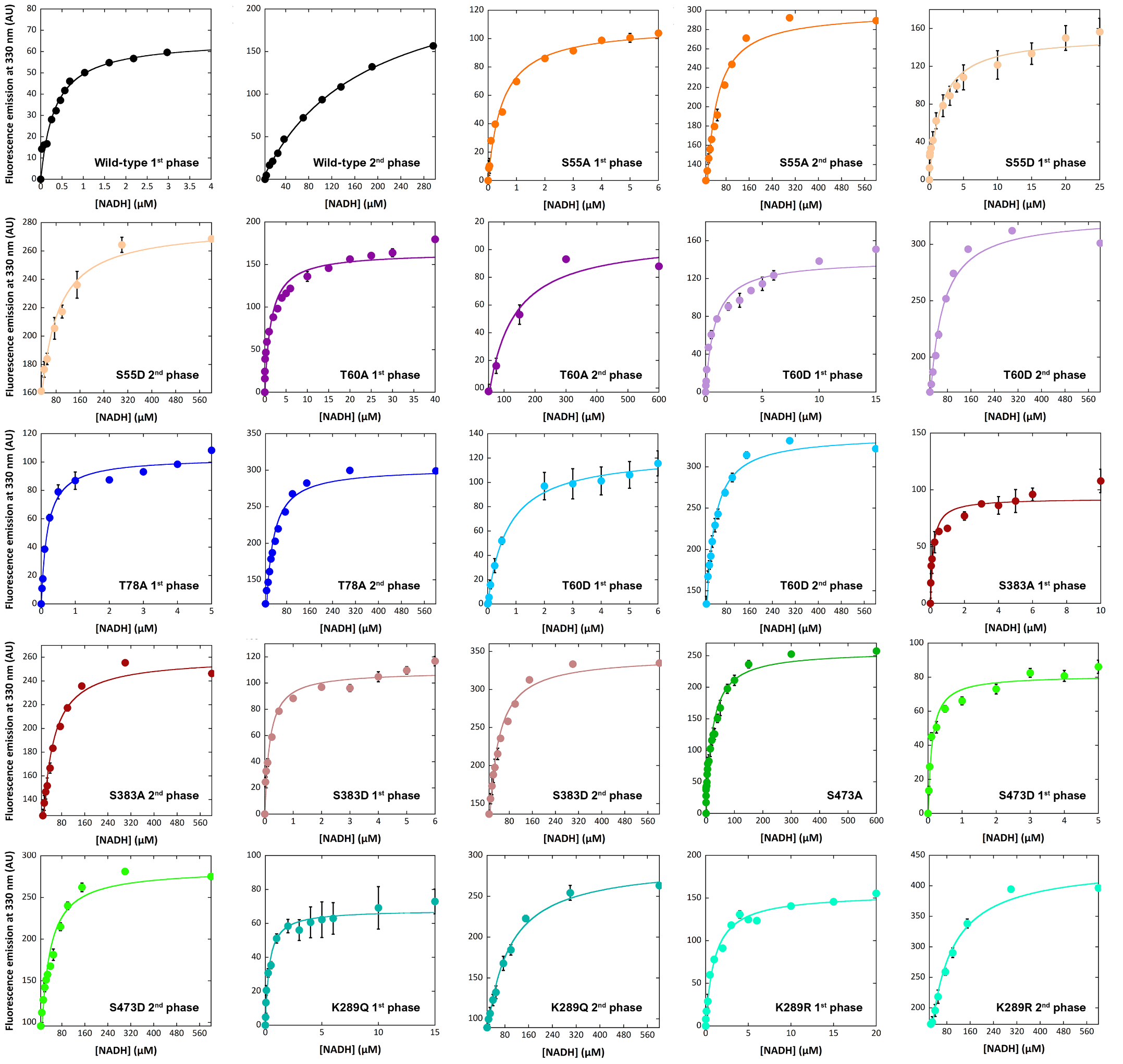


Titration of fluorescence emission at 330 nm for 1 µM PHGDH wild-type and variants in the presence of increasing NADH concentrations, measured in 10 mM potassium phosphate buffer (pH 7.0) at 15 °C: wild-type (black), S55A (orange), S55D (pale orange), T60A (dark purple), T60D (lavender), T78A (blue), T78D (light blue), S383A (firebrick), S383D (salmon), S473A (dark green), S473D (light green), K289Q (dark teal), K289R (aquamarine). Data represent mean ± SD from independent experiments (n = 2).

**
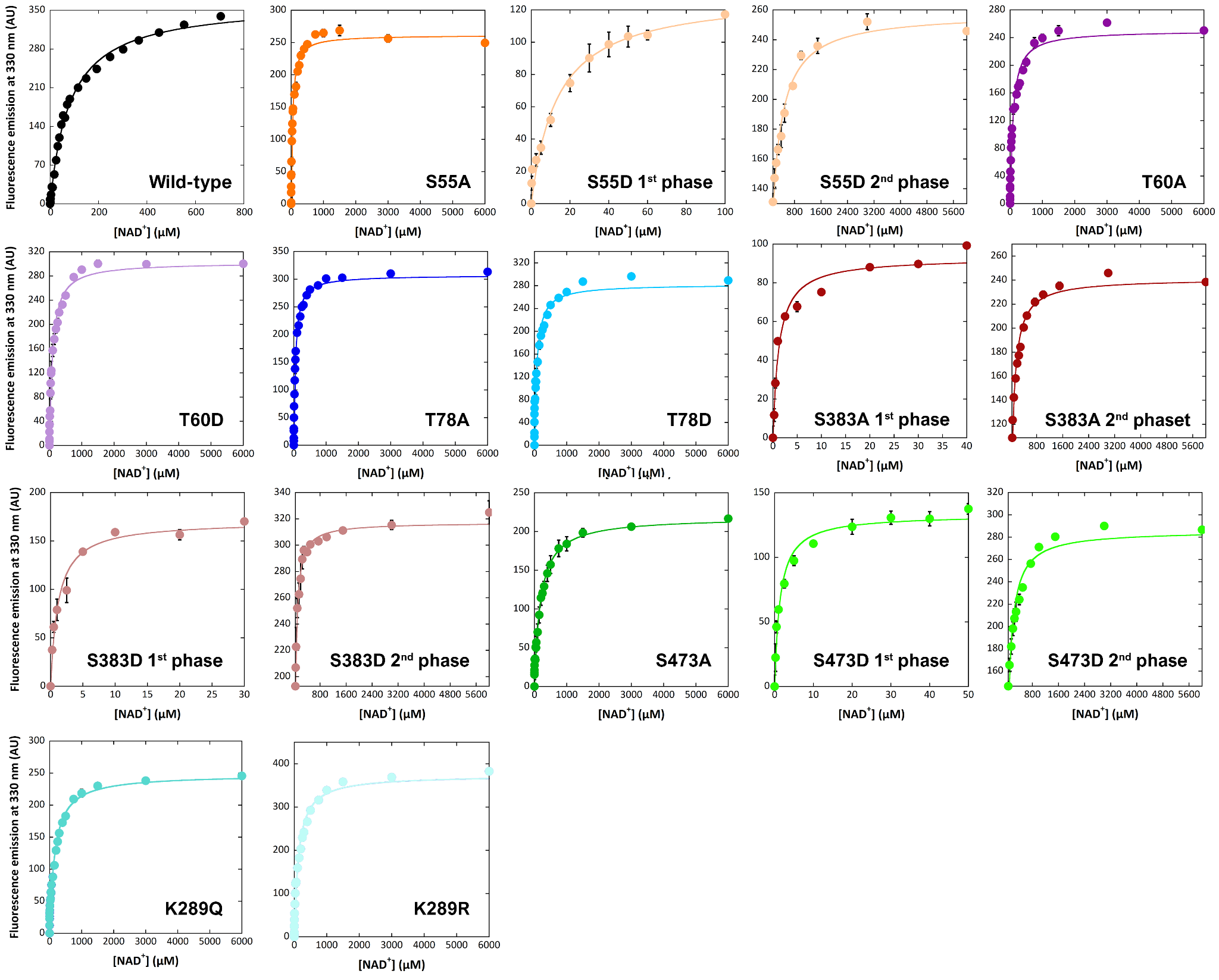
**

**Figure S7. Effect of NAD^+^ binding.**
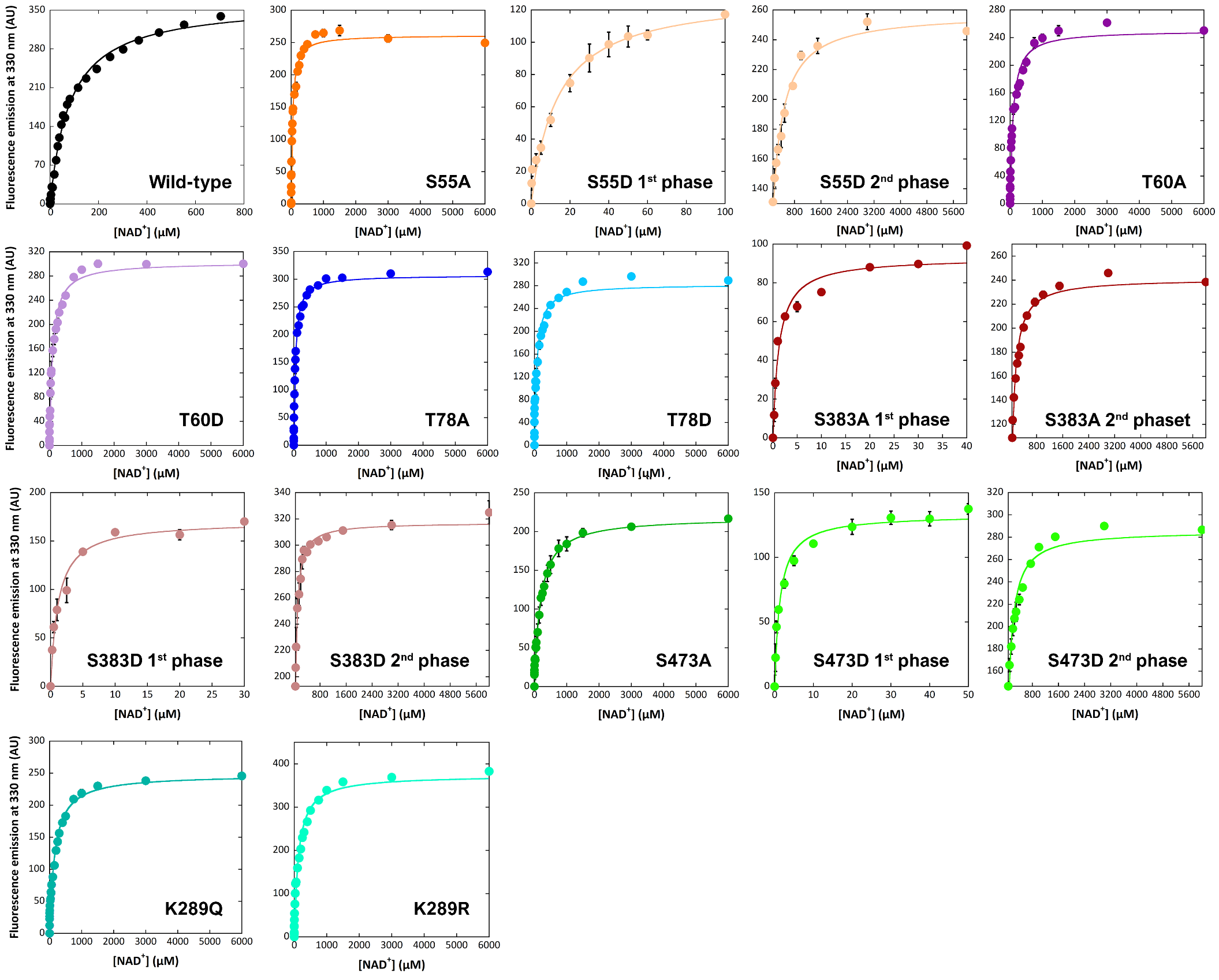


Titration of fluorescence emission at 330 nm for 1 µM PHGDH wild-type and variants in the presence of increasing NAD⁺ concentrations, measured in 10 mM potassium phosphate buffer (pH 7.0) at 15 °C: wild-type (black), S55A (orange), S55D (pale orange), T60A (dark purple), T60D (lavender), T78A (blue), T78D (light blue), S383A (firebrick), S383D (salmon), S473A (dark green), S473D (light green), K289Q (dark teal), K289R (aquamarine). Data represent mean ± SD from independent experiments (n = 2).

**Figure S8. Prediction of subcellular localization of wild-type and variant PHGDH proteins.**
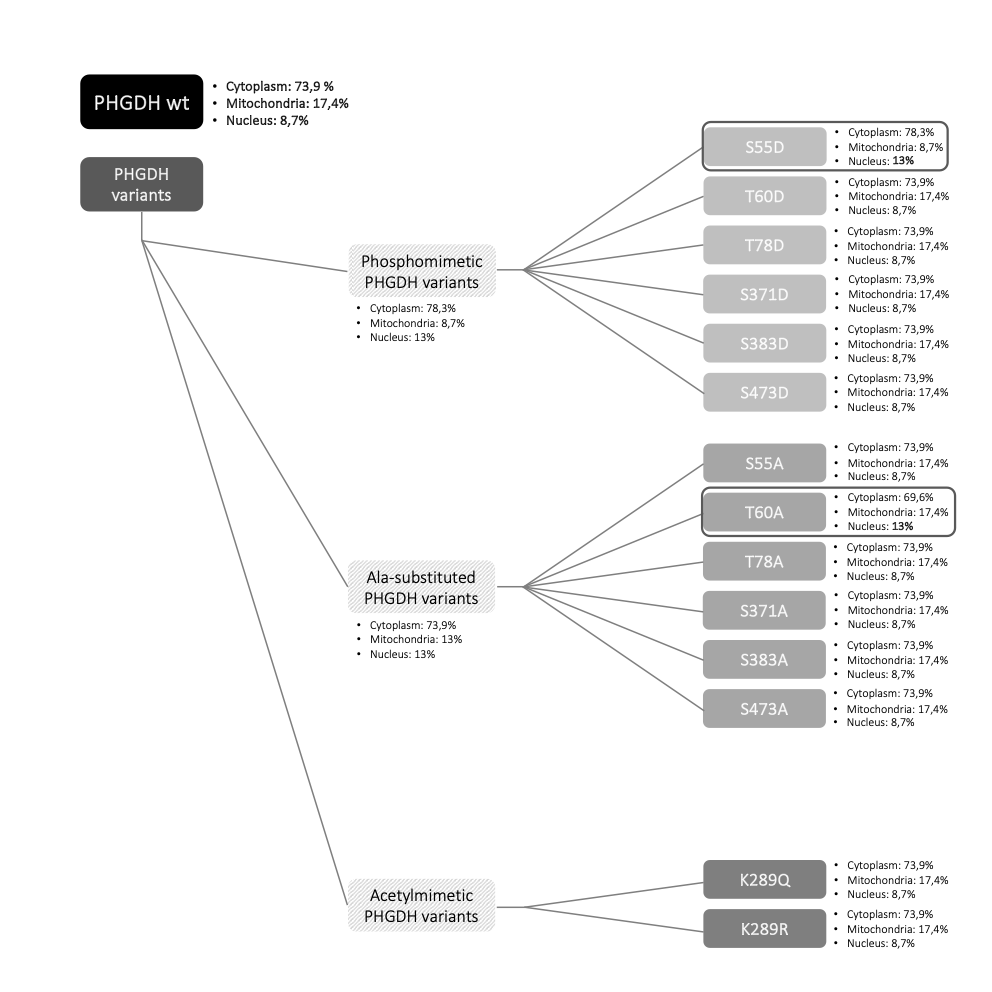


Schematic representation of the PSORT II (<https://psort.hgc.jp/form2.html>) output predicting subcellular localization for wild-type PHGDH and its variants, shown as the percentage distribution across subcellular compartments. For both phosphorylation-mimicking and alanine-substitution variants, analyses were performed in two steps: first, all modified residues were evaluated collectively to assess their cumulative impact on localization; second, each variant was examined individually to identify specific residues contributing to any observed shifts in predicted subcellular distribution.

**
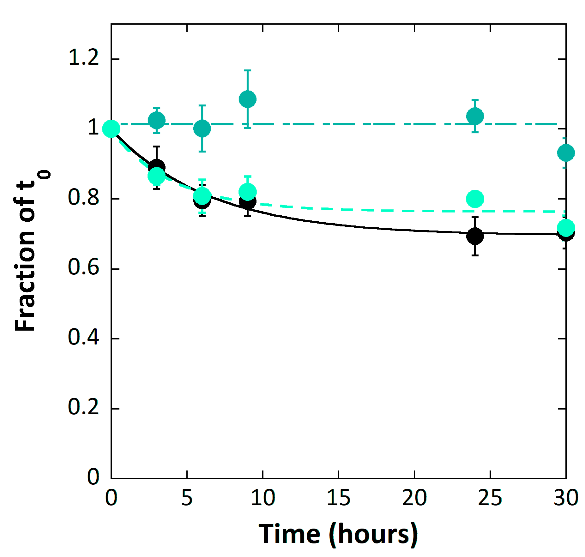
**

**Figure S9. Cycloheximide chase analysis of protein stability in U251 cells**.

Cells overexpressing wild-type (black), K289Q (deep teal) or K289R (aquamarine) PHGDH protein were treated with 75 μg/mL of cycloheximide (CHX) and harvested at the indicated time points. Protein levels were quantified by SDS-PAGE and densitometry, and normalized to the loading control, expressed as fraction of the initial amount (t₀). Data represent mean ± SEM from independent experiments (n = 5).


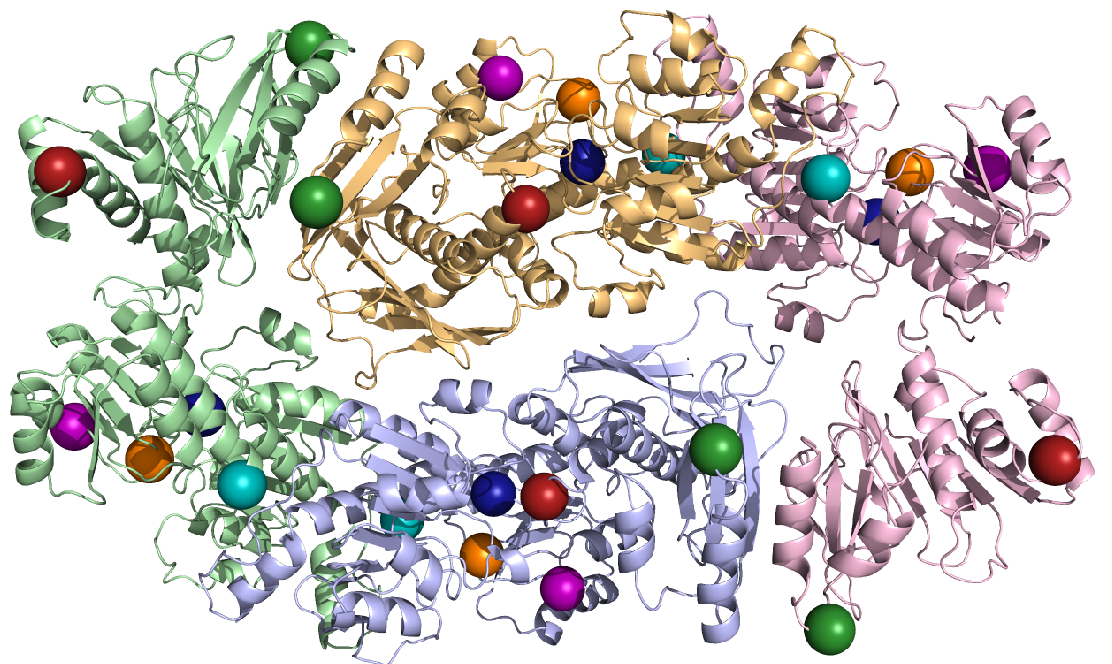


**Figure S10.** **Tetrameric arrangement of the predicted PHGDH structure**.

The model was generated with AlphaFold. Residues analyzed in this study (S55 (orange), T60 (dark purple), T78 (blue), S383 (firebrick), S473 (dark green), and K289Q (deep teal)) are highlighted as spheres.


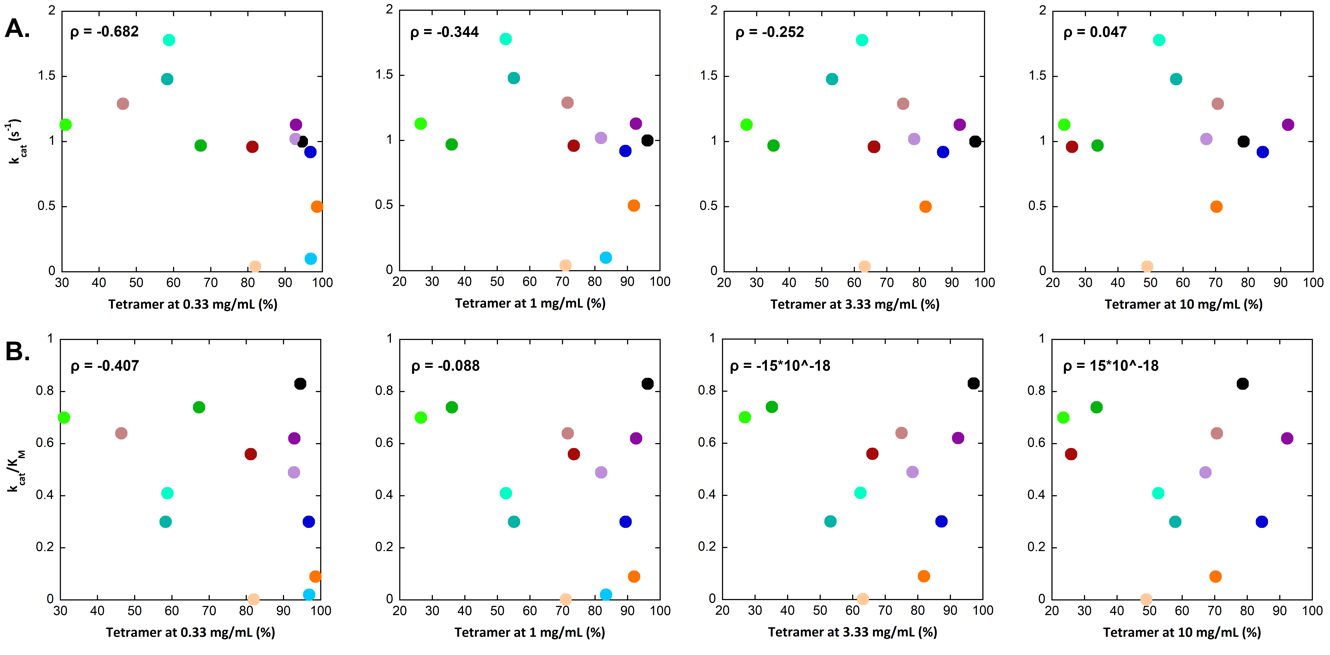


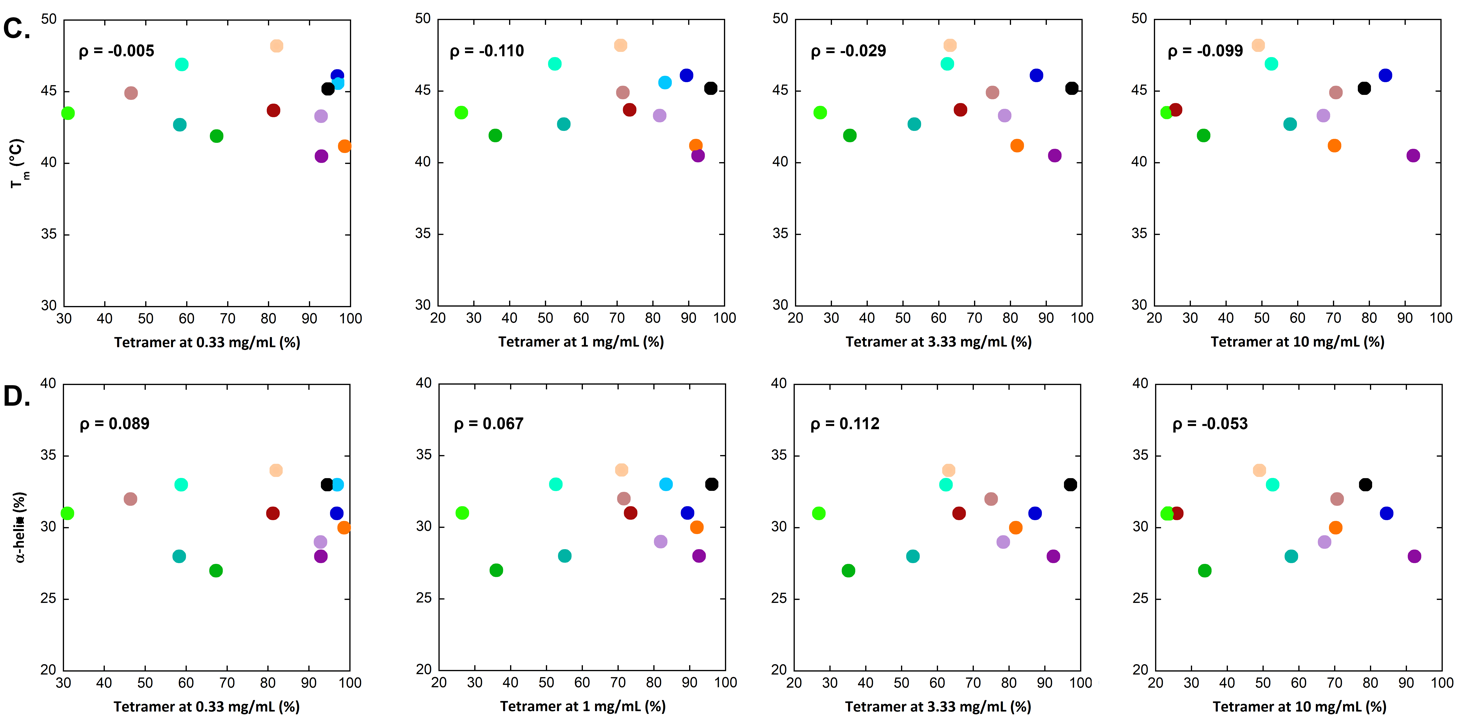


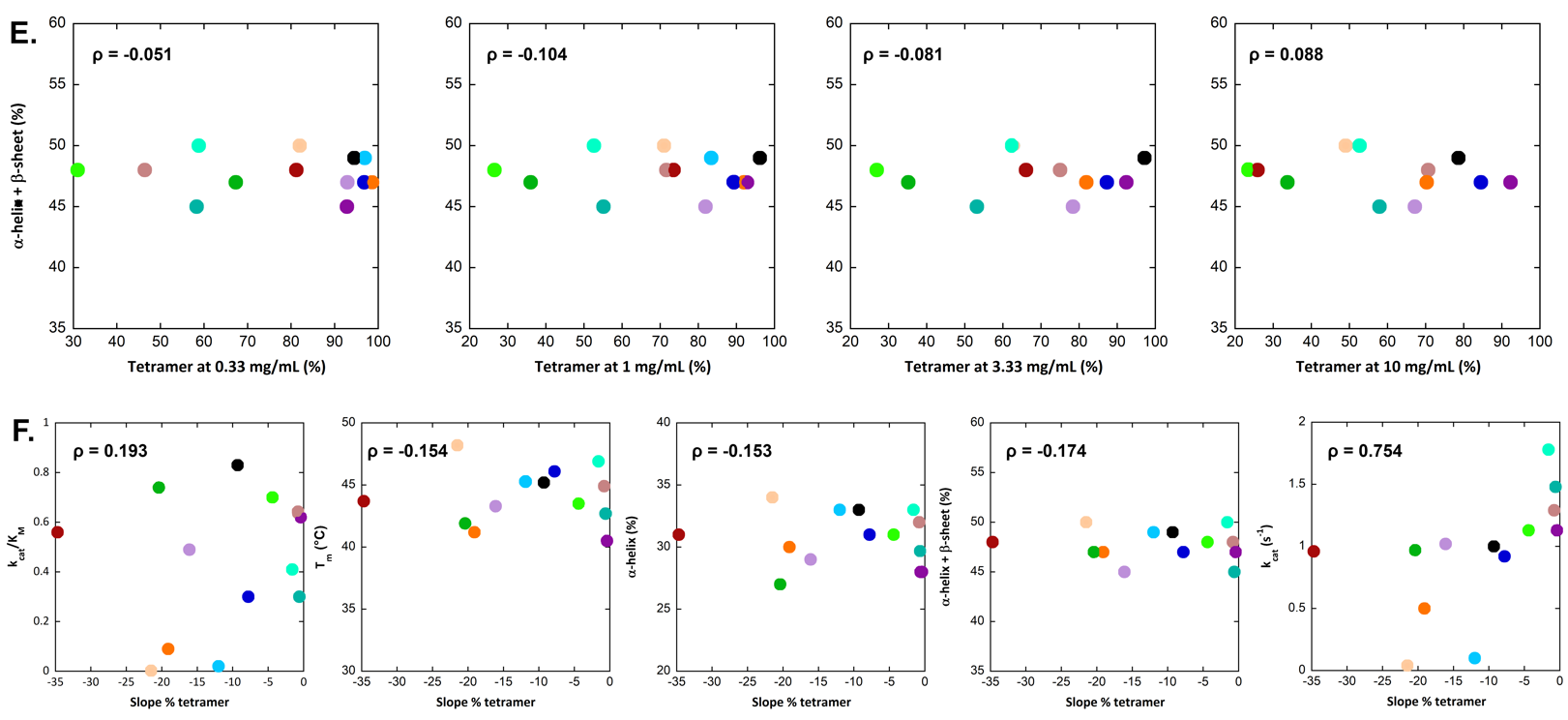


**Figure S11**. **Correlation analyses between PHGDH quaternary assembly and biochemical parameters.**Scatter plots show the relationship between SEC-derived tetramer abundance and biochemical and structural readouts for PHGDH variants (**wild-type**, **S55A**, **S55D**, **T60A**, **T60D**, **T78A**, **T78D**, **S383A, S383D, S473A, S473D, K289Q**, **K289R**; n = 13). Tetramer abundance was quantified at four protein concentrations (0.33, 1, 3.33, and 10 mg/mL) and compared with catalytic turnover (k_cat_, panel A), catalytic efficiency (k_cat_/K_M_, panel B), thermal stability (T_m_, panel C), α-helical content (panel D), and total structured secondary-structure content (α-helix + β-sheet, panel E). Spearman rank correlation coefficients (ρ) are reported for each comparison. No monotonic associations were detected for T_m_ (ρ = −0.005 to −0.110), α-helical content (ρ = −0.053 to 0.112), and α-helix + β-sheet content (ρ = −0.104 to 0.088) across the tested protein concentration range. Catalytic efficiency showed no monotonic association with tetramer abundance at 1–10 mg/mL (ρ ≈ 0), whereas a moderate negative correlation was observed at 0.33 mg/mL (ρ = −0.407). Catalytic turnover (k_cat_) exhibited a stronger negative correlation with baseline tetramer abundance at 0.33 mg/mL (ρ = −0.68), which progressively weakened at higher protein concentrations and was absent at 10 mg/mL. To assess concentration-dependent oligomerization, the slope of % tetramer versus log_10_(protein concentration) (1–10 mg/mL) was calculated and compared with the same parameters (panel F), yielding only weak monotonic associations (ρ = −0.174 to 0.193). No monotonic association was observed between concentration-dependent tetramer loss (slope) and catalytic turnover (ρ = 0.754). All statistical analyses were performed using Spearman’s rank correlation coefficient.
